# Genome-wide association study of diverse soybean [*Glycine max* (L.) Merrill] accessions for agronomic and seed composition traits in Rwanda

**DOI:** 10.1007/s00122-026-05383-7

**Published:** 2026-09-26

**Authors:** Doreen Mutoni, Felix B. Fritschi, Trupti Joshi, Kerry Clark, Jason Gillman, Kristin Bilyeu

**Affiliations:** 1 Division of Plant Science and Technology, University of Missouri, Columbia, MO 65211 USA; 2 Department of Biomedical Sciences, Joan C. Edwards School of Medicine, Marshall University, Huntington, WV 25703 USA; 3 Department of Biomedical Informatics, Biostatistics and Medical Epidemiology, University of Missouri, Columbia, MO 65211 USA; 4 School of Natural Resources, College of Agriculture, Food and Natural Resources, University of Missouri, Columbia, MO 65211 USA; 5 Plant Genetics Research Unit, United States Department of Agriculture, Agricultural Research Service, Columbia, MO 65211 USA

## Abstract

**Key message:**

This research explored the genetic basis of adaptation for soybean to the low-latitude but high-elevation production regions in Rwanda.

**Abstract:**

Soybean [*Glycine max* (L.) Merrill] is an increasingly important crop in Rwanda; however, production remains constrained by limited availability of high-yielding, locally adapted cultivars. The varieties introduced through Rwanda’s Crop Intensification Program were developed for other regions of sub-Saharan Africa and exhibit poor adaptation, delayed maturity and low productivity in Rwanda. This study evaluated diverse germplasm to support development of a national soybean breeding program. A total of 466 accessions representing maturity groups (MG) III–X were evaluated across six environments in Rwanda during 2017 and 2018. Trials were conducted in randomized complete block designs with three or two replications. Traits evaluated included flowering (R1), beginning pod (R3), maturity (R8), reproductive periods, plant height, single-row harvest weight, 100-seed weight and seed protein and oil content. Genotype and genotype-by-environment effects were highly significant (*p* < 0.001) for all traits, with strong correlations among phenology, plant height, reproductive period and seed composition. MG IV–VII accessions demonstrated full season adaptation to Rwanda’s high-elevation environments. Genome-wide association analysis identified significant QTNs: for R1 (30), R3 (26), R8 (33), reproductive periods from R1–R8 (25) and R3–R8 (9), plant height (31), 100-seed weight (30), single-row harvest weight (29), protein (17) and oil (8). GDD-based traits yielded 21, 19, 30, 24 and 10 QTNs for GDD_R1, GDD_R3, GDD_R8, GDD_RP and RP_3, respectively, for 222 unique and a total of 342 QTNs. These findings provide potential markers for selection and a foundation for breeding high-yielding, adapted soybean cultivars for high-elevation, low-latitude agroecosystems.

**Supplementary Information:**

The online version contains supplementary material available at https://doi.org/10.1007/s00122-026-05383-7.

## Introduction

Soybean (*Glycine max*) is a globally significant crop, primarily valued for its high protein content utilized in animal feed and its role as a major source of vegetable oil (FAO 2023). Soybean is a facultative short-day species that originated in the higher latitudes of China approximately 5000 years ago (Carter et al. 2004).

The initiation of flowering in soybean is triggered when day length falls below a genotype-specific critical threshold (Garner and Allard 1920). Genotypes adapted to higher latitudes (long-day environments) tend to enter the reproductive phase prematurely, with limited biomass accumulation, when introduced to lower latitudes (short-day environments) (Watanabe et al. 2012). Conversely, genotypes from lower latitudes often fail to transition to the reproductive phase or exhibit delayed flowering when grown under the extended photoperiods of higher latitudes. Despite this photoperiod sensitivity, soybean has been successfully cultivated across a broad range of latitudes worldwide. Although soybeans are cultivated from approximately 53° N to 35° S, individual genotypes typically exhibit adaptation to relatively narrow latitudinal bands (Zhang et al. 2020).

Adaptation to high-latitude environments has primarily occurred through the selection of null or hypomorphic mutations in maturity genes responsible for photoperiod perception, resulting in reduced photoperiod sensitivity which enables flowering under long-day conditions. In contrast, genotypes adapted to lower latitudes have evolved alleles that delay flowering even under short-day conditions, a phenomenon referred to as long juvenility (Ray et al. 1995). Latitude-specific adaptation in soybean has been facilitated by diverse combinations of photoperiodic and maturity alleles (Dong et al. 2021, 2023).

Flowering time in soybean is heavily regulated by photoperiod, which is inherently associated with latitude and represents a major constraint on geographic adaptation and grain yield potential. In higher latitudes, early flowering is advantageous for maximizing yield before the onset of adverse weather conditions. In contrast, delayed flowering is preferred at lower latitudes, allowing adequate vegetative growth and timely maturity (Zhang et al. 2020).

To date, numerous major genetic loci have been identified as regulators of flowering time and maturity in soybean. These include *E1*, *E2*, *E3*, *E4*, *E5*, *E6*, *E7*, *E8*, *E9*, *E10*, *E11*, *J* and several quantitative trait loci (QTLs), such as *Tof4* (Time of flowering 4), *Tof5*, *Tof8*, *Tof9*, *Tof11*, *Tof12*, *Tof13*, *Tof16*, *LJ16.1*, *LJ16.2* and *Tof18* (Bernard 1971; Buzzell 1971; Buzzell and Voldeng 1980; Bonato and Vello 1999; Liu et al. 2008; Cober et al. 2010; Kong et al. 2014; Lu et al. 2017; Bu et al. 2021; Dong et al. 2021, 2022; Li et al. 2021; Kou et al. 2022). Of these, *E1*, *E2*, *E3*, *E4*, *Tof5*, *Tof11* and *Tof12* facilitate adaptation to higher latitudes, whereas *J/E6*, *FT2a*, *FT5a*, *Tof16* and *Tof18* contribute to adaptation at lower latitudes (Fang et al. 2021; Gupta et al. 2022; Dong et al. 2023; Li et al. 2024).

*E1, E2*, *E3*, *E4*, *E5*, *E6/J*, *E7* and *E8* were identified through classical genetic analyses, while genotyping and molecular approaches enabled the discovery of *E9*, *E10*, *E11*, *Tof5*, *Tof8*, *Tof9*, *Tof11*, *Tof12*, *Tof13* and *Tof16*. Among these, the *E1* locus exerts the most significant effect on flowering and maturity by repressing the expression of *GmFT2a* and *GmFT5a;* the dominant allele of *E9* induces early flowering, whereas the recessive *e9* allele results in delayed flowering (Nan et al. 2014; Kong et al. 2014).

Photoperiod sensitivity in soybean is also mediated by the allelic status of key genes. Under long-day conditions dominant alleles of *E1*, *E2*, *E3*, *E4*, *E7*, *E8* and *E10* inhibit flowering, while dominant alleles at *E6/J*, *E9* and *E11* promote flowering. *E1*, *E3*, *FT2a*, *E6*/*J*, *Tof11*, *Tof16* and *Tof18* were favored during soybean improvement and selection explaining 75.5% of the flowering time phenotypic variation (Li et al. 2024). The genes *E1*, *E2*, *E3*, *E4*, *E7* and *E8* are particularly important for photoperiod sensitivity, especially in response to variations in light quality under artificially extended day lengths. *E3* and *E4* encode phytochrome A photoreceptors (*GmPHYA3* and *GmPHYA2*, respectively), which perceive light cues to regulate downstream gene expression (Buzzell 1971; Buzzell and Voldeng 1980). Additionally, *Dt1* and *Dt2* genes play critical roles in the initiation of flowering and stem termination (Bernard 1972).

During the adaptation of cultivated soybean to high latitudes with longer day lengths, key flowering inhibitor genes such as *E1, E3, E4, Tof5, Tof11* and *Tof12* accumulated sequence polymorphisms that reduced photoperiod sensitivity, leading to early flowering and enabling cultivation in northern regions (Abe et al. 2003; Cober et al. 1996; Liu and Abe 2010; Xu et al. 2013; Dong et al. 2022, 2021; Li et al. 2021; Lu et al. 2020). Conversely, in the adaptation to low-latitude regions with shorter day lengths, flowering promoting factors such as *E6/J, Tof16, Tof18, FT2a* and *FT5a* were genetically impaired, resulting in extended growth periods that improved soybean adaptability and yield under short-day conditions (Ray et al. 1995; Lu et al. 2017; Yue et al. 2017; Fang et al. 2021; Fudge 2022; Kou et al. 2022; Dong et al. 2021). Among these, *E6/J* is a key locus controlling the long juvenile (LJ) trait, essential for soybean adaptation, and is homologous to Arabidopsis *ELF3*, encoding a component of the evening complex in the core circadian clock (Yue et al. 2017; Lu et al. 2017; Dong et al. 2021; Bu et al. 2021; Fang et al. 2021). Mutations in *E6/J* delay flowering under short-day conditions and can increase yield by 30–50% compared to the wild-type allele (Lu et al. 2017).

Growing degree days (GDD) is a crucial concept for crop growth and development. It is used to estimate the heat accumulation needed for crops, including soybeans, to reach specific growth stages. Information about GDD requirements can be used to predict developmental progress, such as when to expect key development milestones and when to harvest (Akyuz et al. 2017). In some situations, GDD also may be useful to determine optimal planting time windows for soybeans, as they require a specific amount of heat to grow efficiently. Different regions have varying temperatures, so using GDD helps tailor planting decisions based on local climates (Akyuz et al. 2017).

Soybean is both photoperiod and temperature sensitive. Photoperiod modifies flowering induction (Yang et al. 2019; Simon-Miquel et al. 2024) and pod setting and growth, while temperature accelerates crop development (Simon-Miquel et al. 2024). Growing degree day (GDD) accumulation starts immediately after planting. Germination typically begins around 50 GDD and accumulate additional GDDs as the plant grows. During reproductive stages, from flowering to pod fill, the plant requires more heat units. Full maturity, or the point when leaves begin to yellow and fall off, generally requires around 2200–2400 GDDs, depending on the variety of soybean in temperate regions (Akyuz et al. 2017). Understanding GDD requirement for soybean allows growers to select the appropriate maturity group for their location to optimize yield potential; for more precise management practices, GDD can help in making decisions regarding irrigation, pest management and harvest timing.

Rwanda lies close to the equator between 1° 4′ and 2° 51′ south latitude and 28°63’ and 30°54’ east longitude. Rwanda has been reported to experience more irregular and unpredictable rainfall patterns with rainy seasons becoming shorter (Gahakwa 2016).

Soybean is grown as a full season crop in Rwanda with a desired maturity duration ranging from 90 to 125 days to fit into the Rwandan growing season. The production objectives for soybean differ amongst farmers depending on location (Mujawamariya 2012). For example, farmers in Bugesera district in the eastern province grow soybean for household consumption, while those in Kamonyi district in the southern province produce it for income due to the presence of a soybean processing plant  (Mujawamariya 2012). The most widely cultivated varieties of soybean are Peka 6, Sc. Squire, Sc. Sequel Bossier, Ogden, Duiker, 449/16, Soprosoy, Yezumutima, Buki and 1740−2E (Mujawamariya, 2012; Tukamuhabwa, 2016; Niykiza et al. 2024).

Locally available soybean varieties have poor yields of less than 1 ton per hectare (Tukamuhabwa, 2016; Diers and Scaboo, 2019). In large part, this is due to a lack of accessibility to diverse soybean germplasm that is necessary for a successful breeding program and would allow for selection for resistance or tolerance to major constraints such as soybean rust susceptibility (caused by *Phakopsora pachyrhizi*), unfavorable soil fertility and *pH*, unpredictable rainfall and extended periods of drought, and a lack of sustainable agronomic practices.

Adaptation of soybean phenology to environmental conditions in Rwanda is a critical aspect for successful production. In North America, soybean is classified into maturity groups (MG) which are well defined and largely determined by allelic combinations at the *E* loci (Bernard 1971; Buzzell 1971; Buzzell and Voldeng 1980; McBlain and Bernard, 1987; Bonato and Vello 1999; Cober and Morrison, 2010; Langewisch et al., 2017; Miranda et al. 2020; Dietz et al. 2021).

Little is known about soybean maturity genes that are favorable for the Rwandan environment or similar tropical high-elevation and low-latitude climates. There are probably hitherto unknown genes present in the gene pool for imparting adaptation to these growing conditions. In this study, a diverse set of germplasm with varying flowering and maturity durations were evaluated for their performance in six environments across typical soybean growing regions in Rwanda.

## Materials and methods

### Selection of germplasm

A total of 1004 genetically diverse soybean accessions (PI) ranging from MG III to MG X were obtained from the USDA National Plant Germplasm System soybean germplasm collection. The panel captured a large amount of genetic diversity and included accessions from several countries of origin, including China, Russia, Vietnam, North/South Korea, Japan and the USA. The accessions were chosen based on their maturity group and then selected for protein, oil, drought tolerance and yield, and soybean rust resistance identified from the literature.

### Experimental design

In September 2016, this diversity panel of 1004 accessions was grown in 2 locations in Rwanda: Bugesera and Rubona. Fifty seeds were planted in 2.5 m single-row plots with 40 cm spacing between rows.

Bugesera is situated (2° 20′ 50 S, 30° 24′ 26 E) at an altitude of 1347 m above sea level (masl) with humic and haplic ferralsols soils with slightly acidic (4.9–6.7 pH) sandy loam soils. Rubona is located (2° 28′ 53.13" S 29° 46′ 22.49" E) at an altitude of 1706 masl with sandy clay loam soils. Nyagatare is at (1° 25′ 47″ S 30° 17′ 8″ E) with slightly acidic (pH 6) and sandy clay loam at 1377 masl (Verdoodt and Van Ranst 2003).

Of the 1004 accessions, 492 accessions were selected during the 2016/2017 growing season for further evaluation. Selections were based on visual selection in the field and whether accessions matured, set seed and the amount of seed harvested from each plot. Accessions that yielded more were preferred as opposed to accessions that only provided less than 10 seeds per plot since the seed would be required for downstream analysis (Supplemental Table 1).

Of the 492 accessions, there were 2 MG III, 139 MG IV, 93 MG V, 105 MG VI, 21 MG VII, 73 MG VIII, 45 MG IX and 14 MG X. These accessions were grown in a randomized complete block design (RCBD) in three maturity group blocks (Early, Mid and Late) in six environments. In the 2017A, 2017B and 2018A growing seasons, they were grown in Bugesera with three replications per season. For season 2018B, the panel was grown in a RCBD with two replications in three locations, Bugesera, Nyagatare and Rubona, for a total of 6 environments since each location-year was treated as a single environment.

Experiments were planted on April 11, 2017 (2017A), October 5, 2017 (2017B), March 21, 2018 (2018A), October 15, 2018 (2018B_BUG), in Bugesera; sowing was on October 17, 2018 (2018B_NYA), in Nyagatare and October 24, 2018 (2018B_RUB), in Rubona. Each plot consisted of a single, 2.5 m row, with interrow spacing of 0.4 m and in-row plant spacing of 0.05 m. Field management was typical of soybean production regions in Rwanda.

### Phenotypic data collection

Days to flowering was recorded as the number of days from planting until 50% of the plants in a plot reached the R1 stage (Fehr et al. 1971). Days to beginning pod/beginning pod stage (R3, characterized by having at least one 5-mm-long pod on one of the four uppermost nodes of the main stem) was noted when 50% of the plants in a plot reached that stage, and days to maturity (R8) was recorded as the number of days from planting to maturity, when 95% of the pods of plants in a plot exhibited a mature pod color at the R8 stage (Fehr et al. 1971). Plant height and single-row plot harvest weight were also collected. The reproductive period (RP) was calculated as the difference between days to flowering and days to maturity; reproductive period_3 (RP_3) was calculated as the difference between days to beginning podding and days to maturity.

Rainfall, elevation, daily minimum and maximum temperature data were provided by Meteo Rwanda from the closest automated metrological stations to monitor the variability of seasonal climatic conditions at the stations.

At maturity, plots were hand harvested and threshed. Flower and pubescence color was recorded for each field plot and compared to known colors for each genotype. After harvest, seeds were inspected and compared to known hilum and seed coat coloration. Any discrepant plots were excluded from further analysis.

A random sample of 50 g of seed from each plot was analyzed at the Bay Farm Research Facility by the University of Missouri Northern Soybean Breeding Program in Columbia, Missouri, for seed composition. Seed samples were visually assessed for seed coat color, hilum color and seed quality as a measure of quality control. Samples with significant visual differences of the same genotype were excluded from further analysis. The samples were milled into a fine powder using a Perten laboratory Mill 3600, “type 0” grinding disk (Perten Instruments, Hägersten Sweden).

Near-infrared spectroscopy (NIRS) was used to predict concentrations of seed composition traits such as protein, oil, amino acids, fatty acids, sugars, ash and fiber traits in seed samples using a Perten DA 7250 instrument and calibration equations developed by Perten, as described by the manufacturer (Perten Instruments, Sweden).

The calculation of GDD is done by tracking daily temperature data and using a specific formula, namely GDD = (Tmax + Tmin)/2 − T base, where Tmax = maximum temperature of the day, Tmin = minimum temperature of the day and Tbase = base temperature, typically 10 °C for soybean, below which no significant growth occurs (Akyuz et al. 2017).

### Statistical analysis

Balanced data allow for unbiased estimates of interaction effects; therefore, 26 accessions with missing replication data from at least one environment after quality control were removed from downstream analyses. This resulted in a total 466 accessions grown at six environments across Rwanda for further analysis.

Analysis of variance (ANOVA) for the studied traits for each environment was completed using the “aov” function in R with genotype, replication and block nested within replication as fixed effects (version 4.6.1; R Core Team, 2026). Means were separated using Tukey’s honestly significant difference test (Tukey’s HSD).

The variance components were used to calculate the heritability of studied traits. Genetic and environmental variances were extracted from the variance component estimates based on the expected mean squares. For the estimation of the heritability, replications and environment were considered random effects.

The heritability (*H*^*2*^) of each trait was calculated as following (Nyquist and Baker 1991):

$$H^{2} (entry\;mean\;basis) = \frac{{\sigma _{g}^{2} }}{{\sigma _{g}^{2} + \frac{{\sigma _{{ge}}^{2} }}{t} + \frac{{\sigma _{e}^{2} }}{{rt}}}}$$

where ${\sigma}_{g}^{2}$ is the variance among genotypes, ${\sigma}_{ge}^{2}$ is the variance of genotype × environment interaction, ${\sigma}_{e}^{2}$ is experimental error, t is number of test environments and r is number of replications.

### Genotyping and quality control

The genotypic data including 42,080 SNP markers for all 466 accessions was downloaded from Soybase website (http://www.soybase.org, “Soybase browser,” accessed 10/08/2024). The information of these SNP markers was retrieved from the seminal (Song et al. 2013) study in which the Illumina SoySNP50k iSelect BeadChip was used for genotyping. The genotypic data was filtered by removing SNPs not located on any of the 20 chromosomes, as well as those with missing rates >5% or with minor allele frequencies < 5%. A total of 34,948 SNPs across 466 accessions were used in the genome-wide association analysis for the studied traits. TASSEL (Bradbury et al., 2007) was used to determine the rate of linkage disequilibrium decay separately for euchromatic and heterochromatic regions using SoySNP50K data (42,080 SNPs in total) using a sliding scale of 50. Results were plotted. Additionally, TASSEL was used to determine Tajima’s D, genetic diversity expressed as theta (θ) and pi (π).

### Population analysis

The 34,948 SNPs remaining after filtering were used for subsequent analysis. Principal components analysis was performed on SNP data using the “*prcomp”* function the “stats” package and graphed using the “ggplot2” package with *R* software. Nei’s genetic similarity was used to construct a pairwise distance matrix using all polymorphic SNPs (Nei 1972, 1978, 1987). Hierarchical cluster analysis using Ward’s minimum variance produced a linkage dendrogram using the “*dendextend*” and “*circlize*” packages (Ward and Hook, 1963) in *R*. The optimal number of SNP-based clusters was determined using the iterative K-means approach in which the procedure successively increases the number of clusters and measures the goodness of fit based on the Bayes information criterion (BIC). Eleven genotypic-based clusters (GBC) were inferred from the inflection point in the BIC curve.

### Genome-wide association mapping

The least-squares means of the studied traits were used as the phenotype values for 466 accessions averaged by environment and across all environments. For each environment as well as across environments, GWAS analyses were performed with 34,948 SNPs using the Bayesian information and Linkage disequilibrium Iteratively Nested Keyway (BLINK) (Huang et al., 2019) available through the “*gapit 3*” package in R (version 4.4.2; R Core Team, 2024)(Wang and Zhang 2021).

Population structure was accounted for using the first three principal components, while a Bonferroni threshold of 0.05 per number of markers (34,948) was used for the significance level [− log10 (*p*) > 5.84]. A genome-wide association study (GWAS) was performed for simple Mendelian traits (flower color, pubescence color) to validate the analysis. Traits which did not display at least one significant association were discarded from further analysis.

## Results

### Correlation among traits

**Fig. 1:**
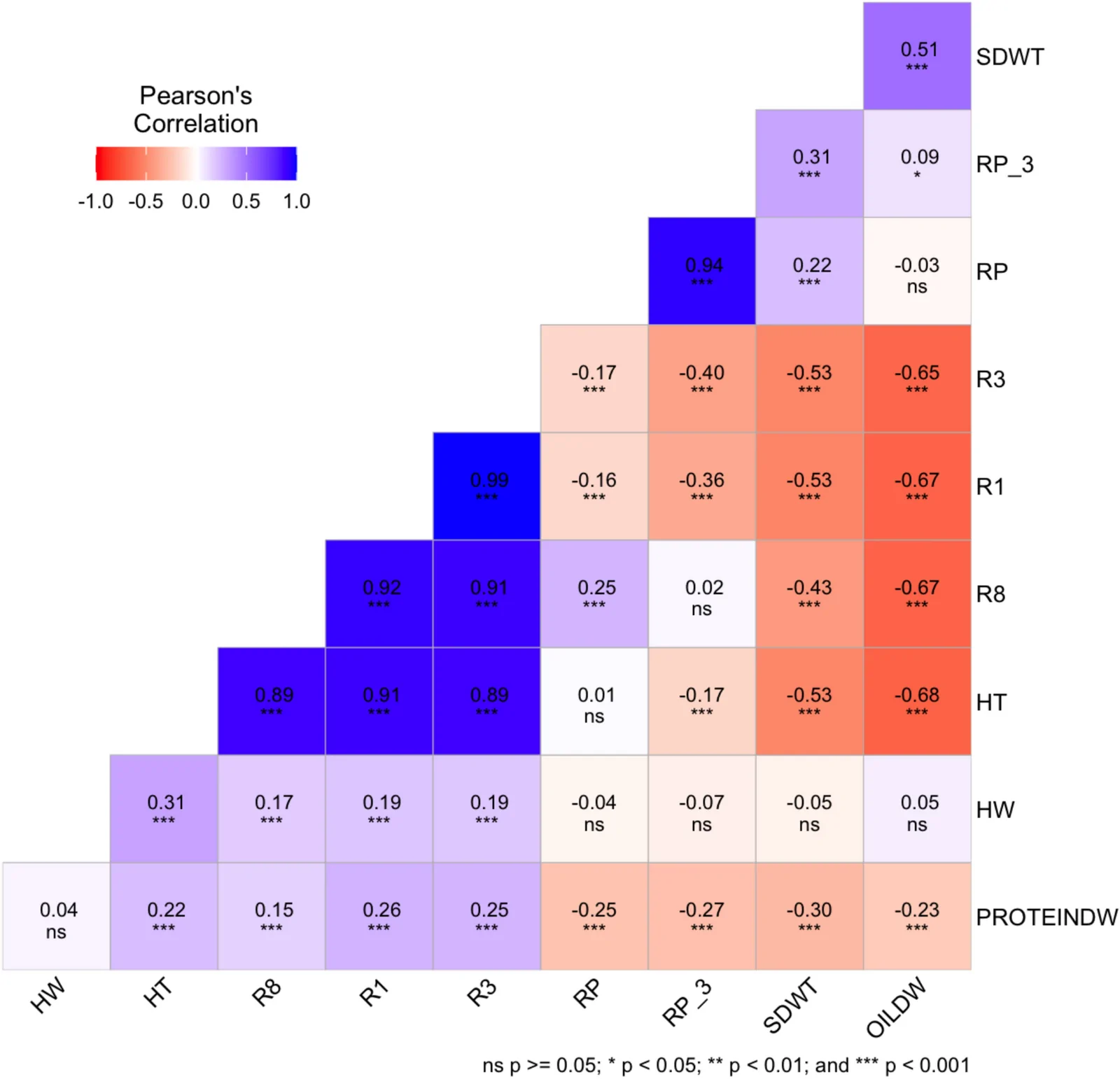
Pearson correlation analysis of R1, R3, R8, plant height, protein and oil traits for 466 soybean genotypes grown across 6 environments. Blue cells indicate positive correlation, orange cells indicate negative correlation, high-saturation colors indicate strong correlation, and low-saturation colors indicate weak correlation. R1 (days to flower), R3 (days to beginning pod stage), R8 (days to physiological maturity), HT (plant height), RP (reproductive period between R1 and R8), RP_3 (reproductive period between R3 and R8), SDWT (100-seed weight), HW (single-row plot harvest weight), OILDW (seed oil dry weight), PROTEINDW (seed protein dry weight)

A correlation analysis was conducted on the seven traits collected as part of this study to determine how traits were related to one another (Fig. 1). Strong positive correlations were observed for all combinations of pairwise analysis among R1, R3, R8, plant height, RP and RP_3 (days to flower, days between beginning pod and physiological maturity) (Fig. 1). Correlation coefficients ranged from 0.89 to 0.99 and were statistically significant (*p* < 0.001), A negative correlation was observed between seed protein and oil content (− 0.23, *p* <  0.001) (Fig. 1). Reproductive period (measured as the days between R1 and R8) was negatively correlated with R1 (− 0.16, *p* < 0.001) and R3 (− 0.17, *p* < 0.001), and positively correlated with R8 (0.25, *p* < 0.001). RP_3 (calculated as the days between R3 and R8), on the other hand, correlated negatively to R1 (− 0.36, *p* < 0.001) and R3 (− 0.40, *p* < 0.001) but was not correlated to R8. The 100-seed weight was positively correlated to oil (0.51, *p* < 0.001), reproductive period (0.22, *p* <  0.001) and RP_3 (0.31, *p* <  0.001), but negatively correlated with protein (− 0.31, *p* <  0.001), plant height (− 0.53, *p* <  0.001), R1 (− 0.53, *p* <  0.001), R3 (− 0.53, *p* <  0.001) and R8 (− 0.43, *p* <  0.001). The single-row plot study design did not allow confident measurement of actual seed yield; the weight of the harvested seed per plot [single-row harvest weight (HW)] was measured and included in the analysis because no other multi-environment data is known to exist for soybean performance in Rwanda. The HW was most positively correlated with plant height (0.31, *p* < 0.001).

There was no marked difference between the Pearson correlation analysis performed using day counts compared to using growing degree data (data not shown).

### Linkage disequilibrium and genetic diversity of the GWAS panel

The range of phenotypic and genetic diversity encompassed by the 466 accessions demonstrates the considerable diversity captured within this GWAS panel (Supplemental Fig. 1). TASSEL was used to analyze linkage disequilibrium decay and genetic diversity (Tajima’s D, as well as theta, and pi, on a per basis). Tajima’s D was found to be 4.4, which as a positive value, indicates genetic bottlenecking was observed throughout the panel and indicates that rare alleles were observed at a low frequency. The estimated mutation rate *theta,* (θ) was found to be 0.36, indicating genetic diversity was present among the panel. Finally, the average pairwise divergence *pi,* (π) was found to be 0.15, indicating there was genetic variation between individuals within the panel. The phylogenetic tree displayed that the accessions used in this panel congregated into five groups. For this analysis, ten *Glycine soja* accessions were added as an outgroup to generate the phylogenetic tree. The *Glycine soja* accessions did not separate out from the other *Glycine max* groups showing that the diversity panel was adequately genetically diverse. The GWAS panel did not otherwise include *Glycine soja* accessions.

Linkage disequilibrium was observed to decay in euchromatic regions to half its total value (0.22) at a distance of 227,924 base pairs (bp) (Supplemental Fig. 2A). In heterochromatic regions, LD decayed to half its total value (0.23) at a distance of 5,396,460 bp (Supplemental Fig. 2B).

**Fig. 2:**
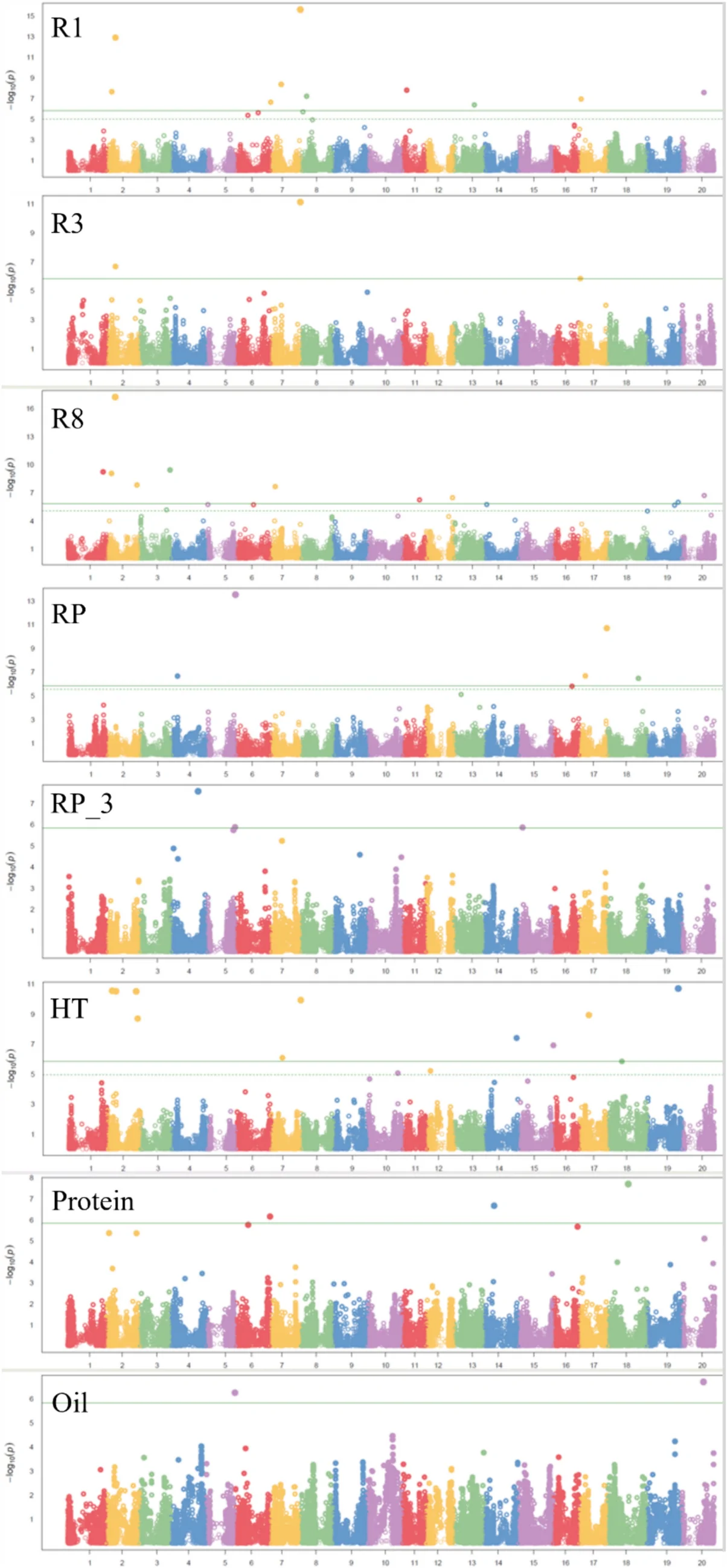
Across All Environments Manhattan plots showing results of the GWAS using BLUPs for phenotype means for the 466 accessions across maturity groups III to X for several traits (R1, R3, R8, RP, RP_3, HT, protein and oil). Green threshold line set at [− log10 (*p*) >5.84]

### Phenotypic variation

The analysis of agronomic traits of 466 diverse soybean genotypes grown in six Rwandan environments indicated significant (*p* < 0.001) genotype, environment and genotype × environment interaction effects for all phenology traits (R1, R3, R8, RP and RP_3), as well as growing degree days (GDD), plant height, harvest weight, 100-seed weight, protein and oil, with the exception of GDD_RP_3 in the Early test, for which the genotype × environment interaction was not significant (Supplemental Table 2). The measured traits were days to flowering (R1), days to beginning pod (R3), days to maturity (R8), reproductive period (RP), the period between R3 and R8 (RP_3), growing degree days (GDD) for each stage, plant height, harvest weight, 100-seed weight (SDWT), protein and oil. A pooled multi-environment analysis of variance (ANOVA) for all traits evaluated in this study is presented in Supplemental Table 2.

The broad-sense estimated heritability (*H*^2^) across the Early, Mid and Late tests ranged from 0.93 to 0.96 for days to flowering (R1), 0.96 to 0.97 for days to beginning pod (R3), 0.73 to 0.88 for days to maturity (R8), 0.95 to 0.95 for seed weight (SDWT) and 0.90 to 0.95 for plant height (HT) (Supplemental Table 2). Heritability for the reproductive period traits was lower, ranging from 0.54 to 0.67 for RP and 0.49 to 0.67 for RP_3 across the three tests. Heritability for harvest weight ranged from 0.75 to 0.84 across the Early, Mid and Late groups. Overall, heritability for phenology traits and plant height ranged from 0.49 to 0.97, with the lowest estimate observed for RP_3 in the Mid group (0.49) and the highest estimate observed for R3 in the Late group (0.97). Seed weight showed consistently high heritability across maturity groups, ranging from 0.945 to 0.949. The heritability for protein in the Early, Mid and Late tests was 0.86, 0.87 and 0.90, respectively, while the heritability for oil in the Early, Mid and Late tests was 0.92, 0.81 and 0.93, respectively. These heritability estimates indicate that genetic differences contributed substantially to variation in protein and oil content across the three test groups. The coefficient of variation for single-row plot harvest weight was relatively large (> 30), ranging from 34.1% in the Early test to 43.0% in the Late test. However, single-row plot harvest weight data are shown because there is no prior information on USDA-sourced soybean germplasm grown in Rwanda; these data may serve as a baseline for future research efforts.

## Genome-wide association study

### Phenology

**Fig. 3:**
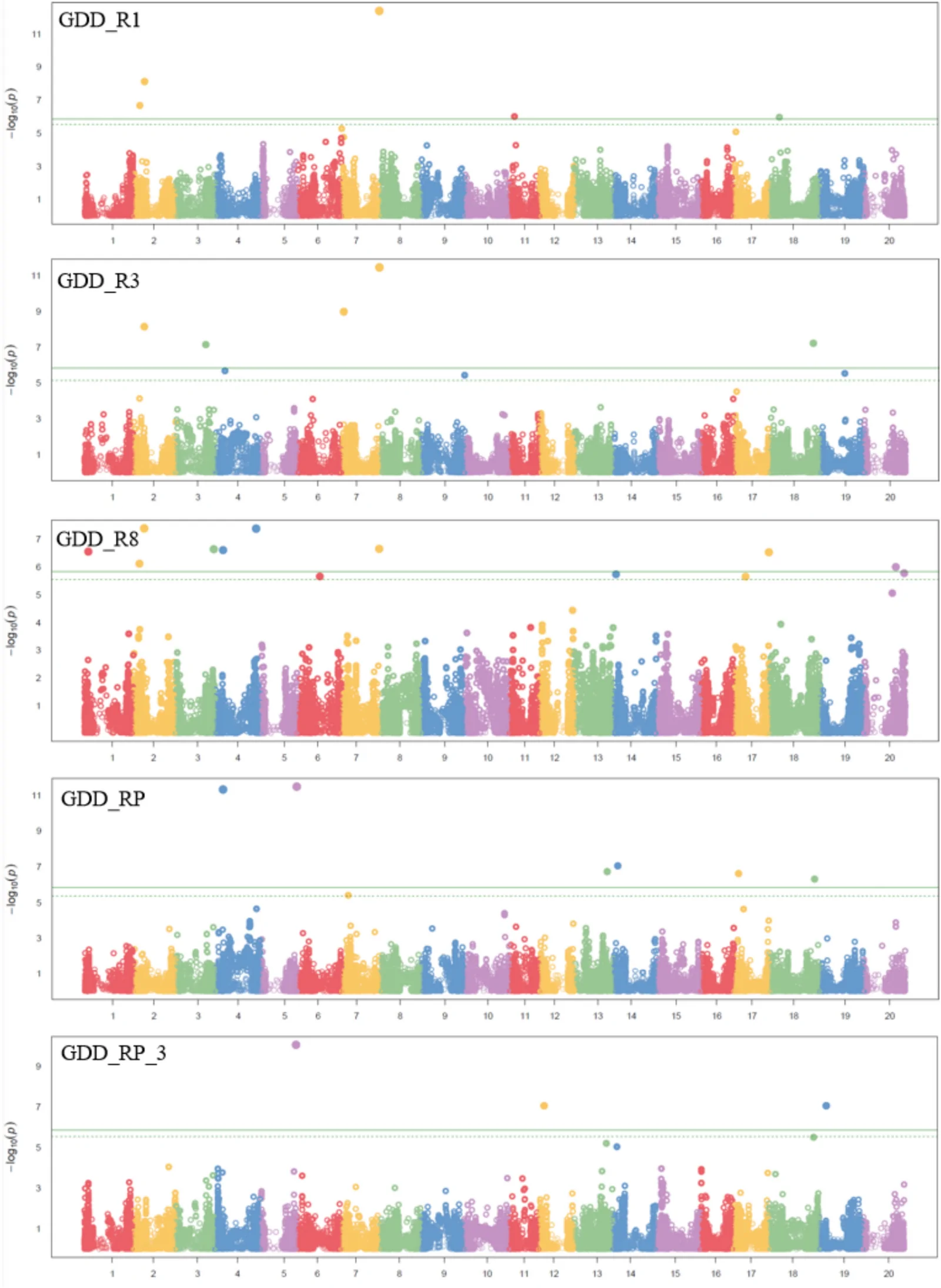
Growing degree day Manhattan plots showing results of the GWAS using BLUPs for phenotypes for the 466 accessions across maturity groups III to X for several traits (R1, R3, R8, RP and RP_3) across all environments utilizing growing degree days. Green threshold line set at − log10 (*p*) >5.84]

The GWAS identified 238 genomic quantitative trait nucleotides (QTN) associated with phenology, plant height, and seed protein and oil traits (Supplemental Table 3 and Supplemental Figs. 3 and 4)). GWAS for days to flower R1, R3 and R8 as well as their growing degree day (GDD) counterparts was carried out. Four regions in total showed pleiotropic effects among phenology traits (R1, R3 and R8) and height or between regular days and growing degree day analyses: two on chromosome (Chr) 2, one on Chr 7 and one on Chr 17 (Figs. 2 and 3). The previously characterized maturity genes *E1*, *E2, E9, Tof9, Tof11* and *Tof12* were also revealed as in this analysis, as was the plant architecture gene *Dt1*.

Thirty quantitative trait nucleotides (QTN) were identified on 14 chromosomes for R1 (days to flowering), 26 QTN on 11 chromosomes for R3 (days to beginning pod stage), 33 QTN on 17 chromosomes for R8 (days to full maturity), 25 QTN on 14 chromosomes for reproductive period (RP) and 9 QTN on five chromosomes for RP_3 (days between beginning pod and physiological maturity) (Supplemental Table 3). Thirty-one QTN were identified on 13 chromosomes for plant height, 30 QTN on 16 chromosomes for 100-seed weight, 39 QTN on 15 chromosomes for single-row harvest weight. Further, 17 QTN were identified on 10 chromosomes for seed protein and 8 QTN on seven chromosomes for seed oil (Supplemental Table 3).

**Fig. 4:**
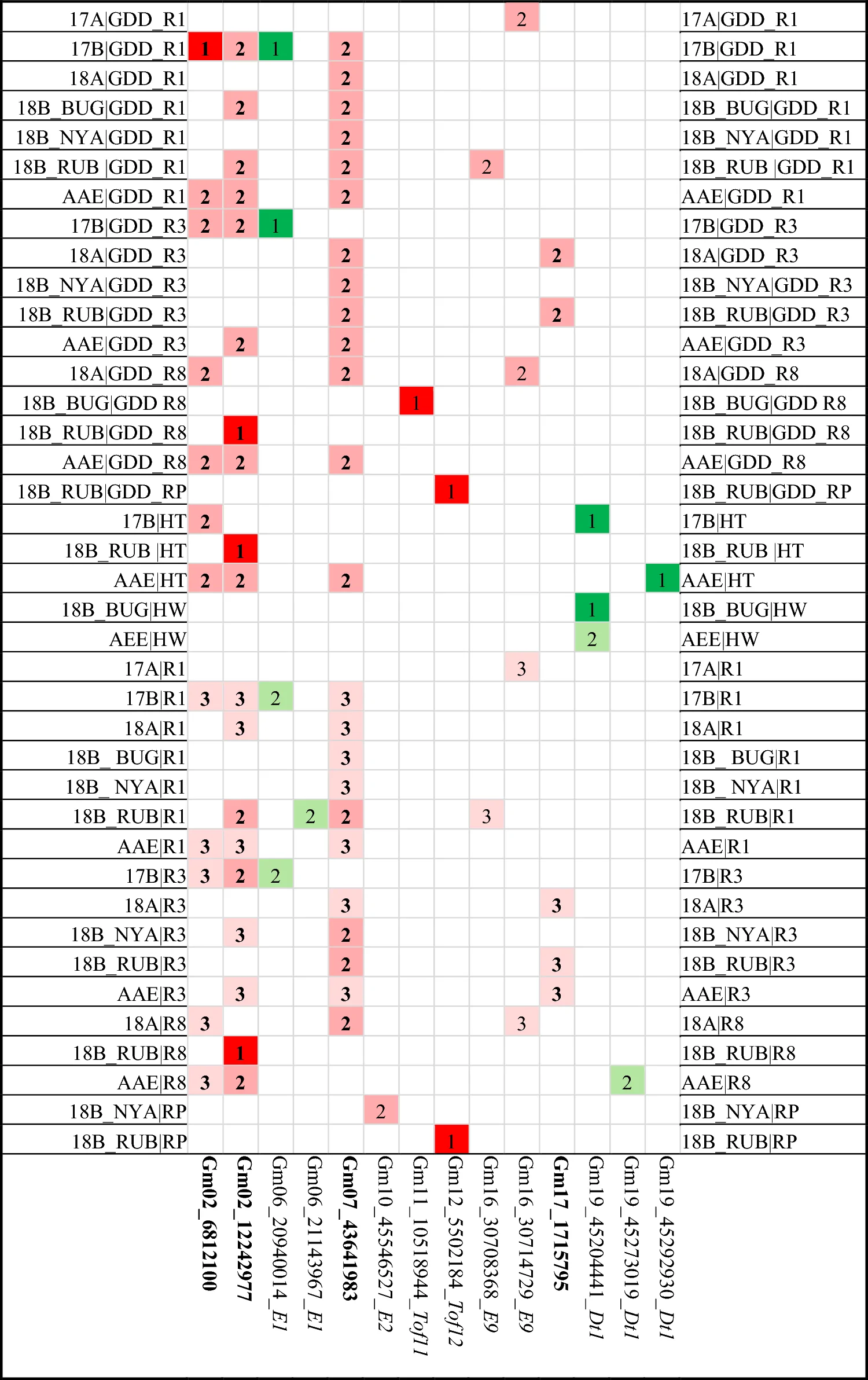
Marker trait associations for R1, R3, R8, plant height (HT), reproductive period (RP), growing degree day R1(GDD_R1), GDD_R3, GDD_R8 and single-row harvest weight (HW) across six environments (17A, 17B, 18A, 18B_BUG, 18B_NYA, 18B_RUB; AEE is across all environments). Red boxes denote negative effects while green boxes indicate positive effects. The relative magnitude of each allelic effect in days, GDD, centimeters or grams was broadly categorized by absolute value rank depicted inside the box (rank 1 darkest shading, rank 2 medium shading and rank 3, light shading). Effect details, − log10 (*p*) values and PVE details are included in Supplemental Table 3

Pleiotropic QTN for phenology and plant height traits from individual environment analyses point to known and unknown genes that influence adaptation to the Rwandan environments (Fig. 4). The maturity genes *E1*, *Tof11* and *Tof12* as well as the plant architecture gene *Dt1* were revealed in this analysis in association with the causative mutations (Škrabišová et al. 2022). *E2* had a negative allelic effect in the 18B_NYA environment for reproductive period that was greater than the positive effect of *E1* observed. *Dt1* had a positive allelic effect for R8, plant height and harvest weight across all environments.

Two major pleiotropic QTN impacting several traits were identified on chromosome 2 (Fig. 4). The SNP ss715583777_T/C at 6,812,100 (Williams 82 reference genome Wm82.a2.v1) was associated with R1, R3, R8 and plant height and explained 11.1%, 2.5%, 1% and 8.7% of the phenotypic variation, respectively (Supplemental Table 3). The alternate variant nucleotide (C) had an allelic effect of − 1.2, − 0.9, − 1.2 and − 2.5: accessions with the alternate genotype flowered earlier by 1.2 days, reached beginning pod stage (R3) and reached maturity approximately one day earlier than accessions with the reference genotype at that position. Accessions with the alternate genotype for ss715583777 were approximately three centimeters shorter overall. In total, 244 accessions had the W82 reference allele, while 219 accessions had the Alt allele.

The SNP ss715581055_T/G located 12,242,977 on Chr 2 affected R1, R3, R8 and plant height where it explained 9.8%, 5.1%, 18.9% and 17.4% of the phenotypic variation, respectively (Fig. 4 and Supplemental Table 3). Overall, the SNP ss715581055_T/G had a greater genotype effect than SNP ss715583777_T/C. Accessions with the W82 reference genotype (T) at ss715581055_T/G on average flowered three and half days earlier than accessions with the alternate genotype (G). Accessions with this reference variant also resulted in reaching the R3 stage and maturity approximately five days earlier and were shorter by approximately eight centimeters. In total, 296 accessions had the W82 allele, while 167 accessions had the Alt allele.

The SNP ss715598192_A/G variant on Chr 7 at 43,641,983 affected R1, R3, R8 and plant height and explained 11.1%, 16.1%, 1% and 8.2% of the phenotypic variation, respectively (Fig. 4 and Supplemental Table 3). The ss715598192 genotype was significant in five of the six environments. The SNP had a genotypic effect of − 1.9, − 2.3, − 2.1 and − 3.6 accessions with that allele reached start of flowering, beginning pod stage (R3) and maturity approximately one-two days earlier than accessions with the reference genotype at that position. This variant also associated with accessions being approximately three centimeters shorter overall. In total, 368 accessions had the W82 reference allele, while 94 accessions had the Alt allele.

The SNP ss715626298_A/G on Chr 17 at 1,730,391 affected R1 while the ss715626293_C/T on Chr 17 at 1,715,795 affected R3, explaining 1.2% and 0.9% of the phenotypic variation, respectively (Fig. 4 and Supplemental Table 3). These SNPs had an allelic effect of − 1.6 and − 0.9, respectively; accessions with those variants flowered and reached beginning pod stage (R3) approximately one day earlier than accessions with the alt allele. In total, 363 accessions had the W82 reference allele, while 100 accessions had the Alt allele.

### Days to flowering (R1)

For the R1 (days to first flower) trait, 30 QTN reached the Bonferroni threshold for the genome-wide association (Supplemental Table 3). There were 16 QTN that had an early flowering allelic effect ranging from 3.2 days to 0.8 days, while eleven QTN had a delayed flowering allelic effect ranging from 0.8 to 2.7 days. Two of the QTN on Chr 6 (ss715593866 and ss715593873) are 1 Mbp upstream to 2 Mbp downstream and in association with the causative mutation for the maturity gene *E1, Glyma.06g207800* with reference to the Williams 82 sequence (Glyma.Wm82.a2) (http://www.soybase.org, “SoyBase browser,” accessed 04/12/2025; Škrabišová et al. 2022). The SNP ss715598192_A/G on Chr 7 at 43,641,983 was significant in five of the six test environments, having an allelic effect of reducing the days to flowering by 1.9 days. Of the 30 significant QTN identified for the R1 GWAS, only two SNPs on Chr 2 have been found in the literature to date. They are SNP ss715583777_T/C at 6,812,100 and SNP ss715581055_T/G located 12,242,977 (Zimmer et al. 2021). The SNP ss715581055_T/G located 12,242,977 was significant in three of the six test environments having an allelic effect of reducing the days to flowering by 2.7 days.

### Days to beginning pod (R3)

There were 26 QTN identified as associated for R3 (days to beginning pod stage) (Supplemental Table 3). 19 QTN had an early podding allelic effect ranging from 2.4 days to 0.8 days, while seven QTN had an allelic effect that delayed R3 ranging from 0.8 to 1.2 days. SNP ss715583777_T/C at 6,812,100 and SNP ss715581055_T/G located 12,242,977 both located on Chr 2 were significant within the 17B environment and across all environments (AAE). SNP ss715598192_A/G on Chr 7 at 43,641,983 was significant in three of the six test environments specifically 18A, 18B_NYA and 18B_RUB having an allelic effect of reducing the days to beginning pod by 2.3 days but was not significant in the 17A, 17B, 18B_BUG growing environments. Of the known maturity genes only the maturity gene *E1* was identified in the R3 GWAS results, having a delay allelic effect of 1.7 days and accounting for 6% of the phenotypic variation observed.

### Days to maturity (R8)

GWAS analysis identified 33 significant QTN for R8 (days to full maturity) (Supplemental Table 3). 23 QTN had an early maturing allelic effect ranging from 4.3 days to 1.0 day, while 10 QTN had an allelic effect that delayed R8 from 1.0 to 3.4 days. An associated region for R8 corresponding to ss715582639 on chromosome 2 near 40.7 M bp appeared to be a model artifact of signal splitting (Supplemental Table 3).

Of particular note was the recently reported *Tof12* gene that encode the pseudo-response regulator (*PRR7b)* protein (Lu et al. 2020). In the present study, QTN close to and in association with the causative mutation for the *Tof12* (Chr 12) allelic effect reduced maturity by 1.9 days and accounted for 6.2% of phenotype variation observed (Škrabišová et al. 2022). Other known loci identified in the results were *E9 Flowering Locus T (FT2a) (Glyma.16G150700)* and *Dt1*, (*Glyma.19G19430).*

### Reproductive period

There were 25 QTN for reproductive period (RP) (days between first flowering and full physiological maturity) (Supplemental Table 3). Significant SNP hits close to the known maturity genes *E2 GIGANTEA (Glyma.10G221500), E8 Cryptochrome 1A (CRY1a;Glyma.04g101500*) and *Tof12* were observed for this GWAS, with an allelic effect of reducing the reproductive period by 2.5, 1.3 and 1.1 days, respectively.

There were 9 QTN significant for RP_3 (days between beginning pod and full physiological maturity) (Supplemental Table 3). Of the 9 QTN identified in this GWAS, three did not overlap with the other full length reproductive period GWAS covering the reproductive period from R1 to R8. Interestingly, one of these three QTN was 1 Mbp downstream of *E1La,* ss715587823 on Chr 4 at 39,484,148, and was significant across all 6 locations, accounting for 3.6% of the phenotype variance explained (PVE%) and resulting in an increase in the reproductive period between R3 and R8 by 1 day.

### Growing degree days

There 104 QTN hits in the growing degree day GWAS results. Of these, there were 21 QTN for GDD_R1, 19 for GDD_R3, 30 for GDD_R8, 24 for GDD_RP and 10 for GDD_RP_3 (Supplemental Table 3). There were significant associations that overlapped between the phenology day GWAS and the respective GDD GWAS. Of these overlapping QTN, the QTNs SNP ss715583777_T/C at 6,812,100 and ss715581055_T/G located 12,242,977 on Chr 2 and SNP ss715598192_A/G variant on Chr 7 at 43,641,983 were significant for the GDD_R1, GDD_R3 and GDD_R8. Also, SNP ss715626293_C/T on Chr 17 at 1,715,795 were significant for GDD_R1 and GDD_R3.

The *Tof 12* (*PRR7b*) SNP ss715613180 located on Chr 12 at 5,502,184 which was significant for the maturity day (R8) and reproductive period GWAS, but was not significant in the GDD_R8 GWAS counterpart; the *Tof 12* (*PRR7b*) SNP ss715613180 was, however, significant for the GDD_RP GWAS. Of the 21 QTN for GDD_R1, five were unique to GDD_R1 GWAS, while the rest overlapped with the days to flowering (R1) GWAS. Of the 19 QTN for GDD_R3, seven were unique to GDD_R3 GWAS and the rest overlapped with the days to beginning pod GWAS. Of the 30 QTN for GDD_R8, 13 were unique to GDD_R8 GWAS, while the rest overlapped with the days to maturity GWAS. Most notable of these were ss715604985_C/T on Chr 9 at 4,655,784 ~ 500 Kilobase pairs (Kbp) upstream of *Tof 9 (Glyma.09G056100)* (Wang et al. 2024) as well as ss715589576_G/A on Chr 4 at 9,078,599 that was ~300 Kbp downstream of *E8 (CRY1a) (Glyma.04g101500)* (Cober et al. 2010)and finally ss715637774_A/G on Chr 20 at 36,907,843 that was ~500 Kbp upstream of *E4 (PHYA2), Glyma.20G090000* (Liu et al. 2008).

### Plant height

For plant height, 31 QTN were identified in each of the test environments (Supplemental Table 3), 11 of which were identified across all environments. Of the 11 significant across all environment markers, the Chr 2 SNP ss715583777_T/C at 6,812,100 was significant within the 17B environment and across all environments (AAE). The SNP ss715581055_T/G located 12,242,977 on Chr 2 was significant in the 18B_RUB environment and across all environments. In addition, the SNP ss715598192_A/G on Chr 7 at 43,641,983 was significant across all environments but not any individual environment, having an allelic effect of − 3.6 cm and accounting for 8.2% of the phenotype variance explained (PVE%). The known stem architecture gene *Dt1* was identified in the 17B environment as well as across all environments.

Based on the SoySNP50k data, 68 of 466 accessions were predicted to have the W82 *E1* reference allele (*e1-as*), while 396 have the Alt allele of a functional *E1.* The distribution of *Dt1* in this study was 216 accessions with the W82 reference allele (dominant indeterminate *Dt1*) and 248 accessions with the Alt determinate *dt1* allele.

### Harvest weight

There were 29 QTN significant for single-row harvest weight, though this measurement is not a substitute for seed yields. There were significant hits near the known genes *Dt1* and *E8 Glyma.04g101500 (CRY1a*) having an allelic effect of 12.3 and 8.4 g, respectively.

### Seed traits

There were 30 QTN significant for 100-seed weight. There were 17 QTN and 8 QTN significant for seed protein and seed oil, respectively. The well-documented locus on Chr 20 that was recently characterized as the *POWR1* (protein, oil, weight, regulator 1) (Goettel et al. 2022; Fliege et al. 2022) was identified in this study for oil but not for protein (Supplemental Table 3).

## Discussion

Soybean as a facultative short-day plant is sensitive to photoperiod and temperature (Garner and Allard 1920; Cober et al. 1996, 2001). As such, accessions introduced from temperate regions and cultivated under low latitudes in tropical and subtropical regions often flower too early resulting in poor yields because of insufficient vegetative growth prior to the reproductive phase of the plant (Dong et al. 2023; Miranda et al. 2020). Soybean varieties insensitive to photoperiod are paramount for the crop to flourish in this environment and the sub-Saharan region at large. The Rwanda soybean growing region poses an adaptability challenge because of its high elevation, cooler temperatures and short days. These data can inform future soybean adaptation for regions of similar climatic conditions, i.e., high elevation and low latitude.

GWASs are powerful tools to search for new loci and causative genic regions for various traits in plants. Several GWASs have been conducted in soybean to date for various agronomic and seed composition traits (Sonah et al. 2015; Bandillo et al. 2015; Fang et al. 2017; Li et al. 2020; Shook et al. 2021; Wu et al. 2023; Hou et al. 2023; Chen et al. 2025). This GWAS is an association study of accessions ranging from Maturity Group III–X of 466 soybeans accessions to mine for key genes in loci related to days to R1, R3, R8, plant height, reproductive period between R1 and R8, reproductive period between R3 and R8 and seed protein and oil content in Rwandan environments. GWAS based on growing degree day (GDD) data for R1, R3, R8 and reproductive period between R1 and R8 as well as reproductive period between R3 and R8 GWAS was also carried out. Of the currently known *E* genes, *E1* was identified in the GWAS for R1, R3, GDD_R1 and GDD_R3. *Dt1* was identified to be significantly associated with R8 and plant height but not for GDD_R8. *E2* was identified to be associated with reproductive period but not its GDD GWAS counterpart.

The genomic region for the *E1* gene (ss715593866, *Glyma.06G207800*) was stable across test environments with a large allelic effect conferred by the dominant *E1* allele on R1 and R3, delaying flowering by 3.2 and 1.7 days, respectively, while increasing the growing degree days by 19.8 and 20.2 for GDD_R1 and GDD_R3, respectively. This result is consistent with the literature where *E1* has been shown to affect time to flowering and maturity. *E1/e1* and *E2/e2* are two major genes that affect time to flowering and maturity that were identified by (Bernard 1971). Dominant alleles of *E1* and *E2* caused delays in flowering, with a stronger effect from *E1* (Bernard 1971). Yamanaka et al. (2005) later fine mapped *E1* with two tightly linked DNA markers, Satt365 (0.1 cM) and GM169 (0.4 cM) and *E1* was eventually positioned to *Glyma.06g207800* by Xia et al. (2012), through positional cloning. *E1* is part of the phytochrome A signaling pathway and downregulates *GmFT2a* and *GmFT5a*, orthologs of *Arabidopsis FLOWERING LOCUS T* responsible for early flowering (Kong et al. 2010).

Of note in this study were two significant hits identified on Chr 2 that had major pleiotropic effects on the traits studied. These two QTN, SNP ss715583777_T/C and SNP ss715581055_T/G had previously been identified (Zimmer et al. 2021). The SNP ss715583777_T/C at position 6,812,100 is located roughly 700 Kb upstream of FT2c, which emerges as a potential candidate gene based on our findings using the GWAS to Genes methodology described in Skrabisova et al. (2022). Wu et al. (2017) demonstrated that the photoperiodic response of *FT2c* mRNA expression in *G. soja* appeared identical to that of *FT2a (Glyma.16G150700)*, a known flowering inducer, suggesting possible functional redundancy. While both *FT2a* and *FT2c* are expressed at extremely low levels under long-day conditions, they exhibited diurnal patterns with a peak at 8 h after dawn under flowering-inductive short days. In contrast, the *G. max* N- and C-terminus *FT2c* (*Glyma.02G069200* and *Glyma.02G069500*) transcripts showed low expression and no obvious photoperiodic response. The domesticated *FT2c* allele typically possesses a ~ 13.6 kb transposon insertion in the third exon (in the reference genome Williams 82). This insertion causes a drastic decrease in *FT2c* mRNA expression. Furthermore, the tissue-specific expression of *FT2c* in *G. soja* also resembled that of *FT2a*, displaying high accumulation in all the examined leaves, but otherwise untraceable in hypocotyls, stems, flower buds, pods and seeds (Wu et al. 2017). The functional divergence (loss of function) in the domesticated *GmFT2c* allele leads to slightly later flowering under short-day conditions compared to the wild allele.

In an experimental setting under flowering-inductive short day, Wu et al. (2017) showed that plants with the homozygous wild *GsFT2c* allele flowered 2.8 days earlier than those with the domesticated *GmFT2c* allele. This late-flowering effect under short days is considered more severe than the effects observed for known major *E* loci or previously reported *FT2a* and *FT5a* QTLs under the same conditions (Wu et al. 2017). The resulting loss of functional *FT2c* may have been favored during early domestication because it contributed to a reduced sensitivity to photoperiods (Wu et al. 2017). This diminished photoperiodic response is essential for the geographic expansion of short-day crops. Our results extend the observations of Wu et al. (2017), suggesting that the *FT2c* region may harbor additional variation contributing to flowering time.

Guo et al. (2024) identified a MADS box gene, one of the four *AP1* homologs as the candidate gene for a flowering time locus (qFT02-2) that was subsequently named *GmAP1d (Glyma.02G121600)*. Only one nonsynonymous polymorphism existed among 1490 predominantly Chinese soybean accessions: at position Chr02:12,087,053. Accessions carrying the Chr02:12,087,053-T allele flowered significantly earlier than those carrying the Chr02:12,087,053-A allele under long-day conditions but not under short-day conditions. A previous study had shown that under short-day conditions, the *AP1* quadruple mutant (*gmap1*) displayed delayed flowering, changes in floral morphology and an increase in node number and node length resulting in plants taller than the wild type (Chen et al. 2020). That study further showed that all four *AP1* homologs were highly expressed in the shoot apex but only *GmAP1d (Glyma.02G121600)* was highly expressed in young pods. *GmAP1d* is approximately 1 Mb away from the SNP ss715581055_T/G located 12,242,977 and warrants further study for the tropics since the Guo et al. (2024) study was restricted to a growth chamber to simulate the short-day conditions.

A WRKY family transcription factor, *Glyma.07G262700* (*GmWRKY75*) is located 100 kb upstream of SNP ss715598192_A/G on Chr 7 at 43,641,983. A recent study revealed that *GmWRKY44* promoted flowering in Arabidopsis by directly upregulating *SOC1* and *LFY*, addressing a critical knowledge gap in the molecular regulation of soybean WRKYs on flowering time and offering a novel candidate gene for optimizing flowering time to enhance soybean yield across diverse agroecological zones (Yu et al. 2025).

Both *Tof11* and *Tof12* that were first reported by Lu et al. (2020) and then Zimmer et al. (2021) were also significant in this study for R8. *Tof12* was also found significant in this study for reproductive period (RP). While *Tof11* was only significant for GDD_R8, *Tof12* was significant for both GDD_RP and GDD_RP3 underscoring the potential for the use of the growing degree day metric in the Rwandan environment. The use of GDD provides a way for accounting for the temperature differences in the environments tested.

*Glyma.17G025700* is a flowering promoting factor 1 located approximately 180 Kbp downstream from the SNP ss715626293_C/T on Chr 17 at 1,715,795 that was significant for GDD R1 and R3. *Glyma.17G014300*, AGAMOUS-like 62, a MADS box transcription factor is located approximately 600 Kbp upstream of SNP ss715626293_C/T.

Growing degree days calculations are predominantly used for corn (*Zea mays*) in the northern states in the USA and Canada. Active Accumulated Temperature (AAT), also known as growing degree days calculation, is most widely used in soybean growing in the main soybean producing regions in China, in northeastern inner Mongolia (Wu et al. 2023). AAT refers to the sum of daily active temperature (when daily average temperature *≥* 10 $^\circ \mathrm{C}$) in a certain period of time or a growing season of a crop. Breeders and farmers in that region use AAT to describe the heat requirement and determine adaptation of each cultivar. Variation of the AAT and its subsequent effect on yield was reported in China (Wen et al. 2022), while a German study reported similar outcomes (Döttinger et al. 2023). Therefore, the use of growing degree days accumulation could be beneficial for soybean breeding in Rwanda and other similar environments of high elevation and low latitude because of the observed temperature difference in the Rwandan soybean growing regions. In addition, this study suggests that the growing degree day metric would be ideal for use in Rwanda because of the lower temperatures in Rwanda compared to other global soybean growing periods. Most notably, the growing degree days calculated in this study were significantly lower than the preferred standard for optimum soybean growth, suggesting that future soybean selections should incorporate individual genotype response to temperature. Studies in temperate regions have shown that GDD range of 2200–2400 are ideal (Akyuz et al. 2017; Katari et al. 2025; Simon-Miquel et al. 2024; Yao and Zhang 2024); however, ours study found significantly lower GDD for soybean maturity (< 2170 GDD). Exploiting this gap could yield great rewards in hastening the breeding process for adapted varieties, especially for the cooler Rubona environment.

Dong et al. (2021) showed that under short-day conditions, *GmLHY1a* also binds the *E1* promoter to influence flowering time. Genetic analysis revealed that while both *Tof16*/*GmLHY1a* and *J* are genetically dependent on *E1*, they control flowering time independently of one another. Further studies have shown that a combination of mutant alleles in *Tof16*, *J* and *E1* is the major genetic basis of soybean adaptation to the tropics (Dong et al. 2021). Among these three loci, our results indicated that only *E1* was significant for days to flowering (R1) and beginning pod (R3). *J* was not detected in our study because accessions carrying the *J* allele were not included at the experimental design stage.

In temperate zones, genes controlling growth and maturity are understood. The *E1*, *E2* and *E3* maturity genes delay flowering when functional and promote early flowering when recessive; recessive alleles of the long juvenile trait (*J*) promote early flowering (Tsubokura et al. 2014; Zhao et al. 2016; Miranda et al. 2020; Dietz et al. 2021). Miranda et al. (2020) showed that in a population of RILs with *j-x* alleles, there was a stepwise increase in days to flower and maturity as functional *E* alleles were added. Our results align with the hierarchy proposed by Miranda et al. (2020)* j* > *E1* > *E2* > *E3* for days to flowering and days to maturity in low-latitude environments. We similarly observed strong effects of *E1* on days to flowering and days to beginning pod, while *E2* was only significant for days to maturity.

*Dt1* is an ortholog of *Arabidopsis thaliana* TERMINAL FLOWER1 (*TFL1*) and specifies an indeterminate stem growth habit, whereas *Dt2* specifies a semi-determinate growth habit (Bernard 1972). MADS box proteins, including *Dt2*, SUPPRESSOR OF OVEREXPRESSION OF CO1 (*GmSOC1*) and MADS box genes downregulated by *E1* (*GmMDE*), repress *Dt1* expression (Xiong et al. 2023). In this GWAS analysis, *Dt1* showed significant associations for several traits: days to maturity, plant height and single-row harvest weight. *Dt2,* however, was not significant in this study. This pattern was consistent with the established genetic hierarchy in which *Dt1* acts as the primary determinant of stem growth habit. *Dt2* influences determinacy indirectly by repressing *Dt1* through interactions with MADS box proteins such as *GmSOC1* and *GmMDE*. Since *Dt2* functions as a modifier rather than a major-effect locus, its impact is smaller and more context-dependent, hence reducing the power of GWAS to detect it. As such, the significance of *Dt1* reflects the biological dominance of *Dt1* in controlling growth habit.

## Conclusion

The characterization of the 466 soybean genotypes included in this study represents a significant step toward advancing the study and utilization of soybean genetic resources. Genome-wide association analysis of traits such as maturity, seed weight and seed composition revealed many markers significantly associated with these characteristics. The identified markers point to potential new candidate genes/regions and will support future efforts to uncover the genetic basis of maturity and seed weight. Of the known maturity loci identified in the study were *E1*, *E2, E4, E8, E9, Tof9, Tof11 Tof12* and *Dt1.* For the Rwandan environments, selecting for the W82 allele at the SNP ss715581055_T/G located 12,242,977 on Chr 2 that affects R1, R3, R8 and plant height would be recommended for future breeding efforts. The SNP ss715583777_T/C at 6,812,100 behaves similarly but has a reduced allelic effect, compared to the SNP ss715581055_T/G and may also be used.

Furthermore, selecting for SNP ss715598192_A/G on Chr 7 at 43,641,983 and SNP ss715626293_C/T on Chr 17 at 1,715,795 could further prove beneficial since accessions with those W82 reference alleles had the highest single-row plot harvest weight though they were short in stature. However, further research with well-designed yield trials would be necessary to determine the utility of these results.

In conclusion, this study provides one of the most comprehensive genome-wide assessments of soybean genetic diversity and trait architecture in tropical African environments. This work bridges the gap between global soybean genomic resources and region-specific breeding needs by linking agronomically important traits to specific genomic regions. Furthermore, the identification of QTN with consistent and pleiotropic effects across traits highlights opportunities for more efficient marker-assisted and genomic selection strategies.

Importantly, the validation of W82-derived alleles under Rwandan conditions offers new insight into the transferability of reference genome-based discoveries to tropical production systems. These findings enhance the understanding of soybean adaptation in tropical environments while also providing actionable tools for accelerating the development of high-yielding, climate-resilient cultivars, with the ultimate goal being to contribute to global efforts in food security and sustainable agriculture.

Ultimately, this work demonstrates how integrating diverse germplasm with genome-wide analysis can unlock underutilized genetic variation and drive the next generation of soybean improvement in emerging production regions.

## Supplementary Information

Below is the link to the electronic supplementary material.

Supplementary file1 (DOCX 153 KB)

Supplementary file2 (DOCX 1061 KB)

## Data Availability

All data supporting the findings of this study are available within the paper and its Supplementary Information.

## Abbreviations

GDD: Growing degree day
GWAS: Genome-wide association study
GoR: Government of Rwanda
HW: Harvest weight
MG: Maturity group
PI: Plant Introduction
RP: Reproductive Period
