# Supplementary material for: Genome-wide association study of diverse soybean [Glycine max (L.) Merrill] accessions for agronomic and seed composition traits in Rwanda
Source: Theor Appl Genet. 2026 Sep 26;139(10):281. doi: 10.1007/s00122-026-05383-7 (PMC13616022; doi:10.1007/s00122-026-05383-7)
Supplement: Supplementary file 1 [file 122_2026_5383_MOESM1_ESM.docx]

Supplemental Table 1. Summary information of 492 Soybean accessions selected for multi-location trials in 2017 and 2018 in Rwanda

| No | **Name** | **MG** | **Country** | **Trait** | **Excluded from 466** |
| --- | --- | --- | --- | --- | --- |
| 1 | PI518671 | III | United States | Rust |  |
| 2 | PI547875 | III | Unknown | Rust |  |
| 3 | FC031685 | IV | Unknown | Oil |  |
| 4 | FC031702 | IV | Unknown | Oil |  |
| 5 | FC033243 | IV | Unknown | Oil |  |
| 6 | PI054614 | IV | China | Phase 1 Seq set |  |
| 7 | PI058955 | IV | China | Phase 1 Seq set |  |
| 8 | PI068011 | IV | China | Oil |  |
| 9 | PI080479 | IV | Unknown | Oil |  |
| 10 | PI080834-2 | IV | Unknown | Oil |  |
| 11 | PI084669N | IV | South Korea | Oil |  |
| 12 | PI088302-2 | IV | China | Oil |  |
| 13 | PI088444 | IV | China | Drought Tolerance |  |
| 14 | PI088459 | IV | China | Oil |  |
| 15 | PI089061-3 | IV | Unknown | Oil |  |
| 16 | PI089772 | IV | China | Phase 1 Seq set |  |
| 17 | PI092651 | IV | Unknown | Phase 1 Seq set |  |
| 18 | PI094159-3 | IV | Unknown | Phase 1 Seq set |  |
| 19 | PI157437 | IV | South Korea | Oil |  |
| 20 | PI170380 | IV | Turkey | Oil |  |
| 21 | PI171428 | IV | China | Phase 1 Seq set |  |
| 22 | PI179826 | IV | China | Oil |  |
| 23 | PI200457 | IV | Japan | Rust |  |
| 24 | PI235344 | IV | Uruguay | Oil |  |
| 25 | PI248402 | IV | United States | Oil |  |
| 26 | PI253665A | IV | China | Oil |  |
| 27 | PI371611 | IV | Pakistan | Oil |  |
| 28 | PI398965 | IV | South Korea | Phase 1 Seq set |  |
| 29 | PI404177 | IV | China | Protein |  |
| 30 | PI407778A | IV | South Korea | Drought tolerance |  |
| 31 | PI407973A | IV | South Korea | Drought tolerance |  |
| 32 | PI417092 | IV | Japan | Drought tolerance |  |
| 33 | PI417345B | IV | China | Phase 1 Seq set |  |
| 34 | PI417479 | IV | Japan | Phase 1 Seq set |  |
| 35 | PI423841 | IV | South Korea | Drought tolerance |  |
| 36 | PI424460 | IV | South Korea | Drought tolerance |  |
| 37 | PI424608A | IV | South Korea | Drought tolerance |  |
| 38 | PI437127A | IV | Georgia | Phase 1 Seq set |  |
| 39 | PI438496C | IV | United States | Phase 1 Seq set |  |
| 40 | PI458020 | IV | South Korea | Drought tolerance |  |
| 41 | PI464912 | IV | China | Phase 1 Seq set |  |
| 42 | PI495020 | IV | China | Phase 1 Seq set |  |
| 43 | PI506937 | IV | Japan | Oil |  |
| 44 | PI506954 | IV | Japan | Oil |  |
| 45 | PI506973 | IV | Japan | Oil |  |
| 46 | PI506992 | IV | Japan | Oil |  |
| 47 | PI507066 | IV | Japan | Drought tolerance |  |
| 48 | PI507180 | IV | Japan | Phase 1 Seq set |  |
| 49 | PI507456 | IV | Japan | Oil |  |
| 50 | PI507467 | IV | Japan | Phase 1 Seq set |  |
| 51 | PI507480 | IV | Japan | Phase 1 Seq set |  |
| 52 | PI508296E | IV | South Korea | Protein |  |
| 53 | PI509088 | IV | South Korea | Protein & Oil |  |
| 54 | PI518668 | IV | United States | Phase 1 Seq set |  |
| 55 | PI518673 | IV | United States | Yield |  |
| 56 | PI525454 | IV | United States | Yield |  |
| 57 | PI536636 | IV | United States | Oil |  |
| 58 | PI540555 | IV | United States | Oil |  |
| 59 | PI543856 | IV | United States | Oil |  |
| 60 | PI548193 | IV | Unknown | Phase 1 Seq set |  |
| 61 | PI548297 | IV | China | Oil |  |
| 62 | PI548309 | IV | China | Oil |  |
| 63 | PI548359 | IV | China | Phase 1 Seq set |  |
| 64 | PI548364 | IV | Japan | Oil&Phase 1 Seq set |  |
| 65 | PI548400 | IV | China | Phase 1 Seq set |  |
| 66 | PI548427 | IV | China | Phase 1 Seq set |  |
| 67 | PI548514 | IV | United States | Oil |  |
| 68 | PI548532 | IV | United States | Oil |  |
| 69 | PI548533 | IV | United States | Oil |  |
| 70 | PI548586 | IV | United States | Oil |  |
| 71 | PI548619 | IV | United States | Phase 1 Seq set |  |
| 72 | PI548626 | IV | United States | Oil |  |
| 73 | PI548633 | IV | United States | Phase 1 Seq set |  |
| 74 | PI548645 | IV | United States | Oil |  |
| 75 | PI559932 | IV | United States | Phase 1 Seq set |  |
| 76 | PI561371 | IV | China | Phase 1 Seq set |  |
| 77 | PI561701 | IV | Unknown | Phase 1 Seq set |  |
| 78 | PI567189A | IV | Vietnam | Rust |  |
| 79 | PI567366B | IV | China | Protein |  |
| 80 | PI567368 | IV | China | Protein |  |
| 81 | PI567398 | IV | China | Drought tolerance |  |
| 82 | PI567415A | IV | China | Phase 1 Seq set |  |
| 83 | PI567434 | IV | China | Oil |  |
| 84 | PI567453 | IV | China | Oil |  |
| 85 | PI567488A | IV | China | Phase 1 Seq set |  |
| 86 | PI567489A | IV | China | Phase 1 Seq set |  |
| 87 | PI567527 | IV | China | Oil |  |
| 88 | PI567561 | IV | China | Drought tolerance |  |
| 89 | PI567580A | IV | China | Protein & Oil |  |
| 90 | PI567604A | IV | China | Phase 1 Seq set |  |
| 91 | PI567610B | IV | China | Protein |  |
| 92 | PI567614C | IV | China | Protein |  |
| 93 | PI567617B | IV | China | Protein |  |
| 94 | PI567641 | IV | China | Protein |  |
| 95 | PI567669 | IV | China | Protein |  |
| 96 | PI567671B | IV | China | Protein |  |
| 97 | PI567675 | IV | China | Phase 1 Seq set |  |
| 98 | PI567685 | IV | China | Phase 1 Seq set |  |
| 99 | PI567689 | IV | China | Protein |  |
| 100 | PI567702B | IV | China | Protein |  |
| 101 | PI567709 | IV | China | Protein |  |
| 102 | PI567710 | IV | China | Protein |  |
| 103 | PI567711A | IV | China | Protein |  |
| 104 | PI567711B | IV | China | Protein |  |
| 105 | PI567717C | IV | China | Protein |  |
| 106 | PI567722 | IV | China | Protein |  |
| 107 | PI567726 | IV | China | Phase 1 Seq set |  |
| 108 | PI567730 | IV | China | Protein |  |
| 109 | PI567734 | IV | China | Protein |  |
| 110 | PI567746 | IV | China | Phase 1 Seq set |  |
| 111 | PI567767B | IV | China | Yield |  |
| 112 | PI567780B | IV | China | Phase 1 Seq set |  |
| 113 | PI574477 | IV | China | Oil&Phase 1 Seq set |  |
| 114 | PI576440 | IV | United States | Yield |  |
| 115 | PI578477A | IV | China | Drought tolerance |  |
| 116 | PI578495 | IV | China | Phase 1 Seq set |  |
| 117 | PI587588A | IV | China | Phase 1 Seq set |  |
| 118 | PI587716B | IV | China | Protein |  |
| 119 | PI587804 | IV | China | Phase 1 Seq set |  |
| 120 | PI590931 | IV | United States | Yield |  |
| 121 | PI590932 | IV | United States | Yield |  |
| 122 | PI592940 | IV | China | Phase 1 Seq set |  |
| 123 | PI594410 | IV | China | Drought tolerance |  |
| 124 | PI602502B | IV | China | Phase 1 Seq set |  |
| 125 | PI602993 | IV | China | Phase 1 Seq set |  |
| 126 | PI603156 | IV | North Korea | Protein |  |
| 127 | PI603162 | IV | North Korea | Protein & Phase 1 Seq set |  |
| 128 | PI603176A | IV | North Korea | Protein |  |
| 129 | PI603487A | IV | China | Protein |  |
| 130 | PI603913D | IV | North Korea | Oil |  |
| 131 | PI605834C | IV | Vietnam | Protein |  |
| 132 | PI605882B | IV | Vietnam | Protein |  |
| 133 | PI605885C | IV | Vietnam | Protein |  |
| 134 | PI606440A | IV | Vietnam | Rust |  |
| 135 | PI614155 | IV | United States | Yield |  |
| 136 | PI614732 | IV | United States | Yield |  |
| 137 | PI614806 | IV | United States | Oil |  |
| 138 | PI632639B | IV | Vietnam | Rust |  |
| 139 | PI632645 | IV | Vietnam | Rust |  |
| 140 | PI632667 | IV | Vietnam | Rust |  |
| 141 | PI639740 | IV | Unknown | Rust |  |
| 142 | FC031683 | V | Unknown | Oil |  |
| 143 | PI062203 | V | China | Phase 1 Seq set |  |
| 144 | PI080466 | V | Japan | Oil |  |
| 145 | PI083942 | V | South Korea | Phase 1 Seq set |  |
| 146 | PI324924 | V | South Africa | Phase 1 Seq set |  |
| 147 | PI342434 | V | Japan | Protein & Phase 1 Seq set |  |
| 148 | PI371610 | V | Pakistan | Oil |  |
| 149 | PI398316 | V | South Korea | Oil |  |
| 150 | PI398589 | V | South Korea | Protein |  |
| 151 | PI398597 | V | South Korea | Protein |  |
| 152 | PI398610 | V | South Korea | Protein |  |
| 153 | PI398612 | V | South Korea | Protein |  |
| 154 | PI398613 | V | South Korea | Protein |  |
| 155 | PI398633 | V | South Korea | Phase 1 Seq set |  |
| 156 | PI398722 | V | South Korea | Protein |  |
| 157 | PI089154S | V | North Korea | Oil |  |
| 158 | PI407892B | V | South Korea | Protein |  |
| 159 | PI407963 | V | South Korea | Protein |  |
| 160 | PI407998D | V | South Korea | Protein |  |
| 161 | PI408012 | V | South Korea | Protein |  |
| 162 | PI408056 | V | South Korea | Protein |  |
| 163 | PI408063 | V | South Korea | Protein |  |
| 164 | PI408098 | V | South Korea | Oil |  |
| 165 | PI408337 | V | South Korea | Oil |  |
| 166 | PI417329 | V | Japan | Oil |  |
| 167 | PI417581 | V | United States | Phase 1 Seq set |  |
| 168 | PI424331 | V | South Korea | Oil |  |
| 169 | PI424398 | V | South Korea | Oil |  |
| 170 | PI438343 | V | Australia | Oil |  |
| 171 | PI458146 | V | South Korea | Oil |  |
| 172 | PI458186 | V | South Korea | Protein |  |
| 173 | PI458192 | V | South Korea | Protein |  |
| 174 | PI458306B | V | South Korea | Oil |  |
| 175 | PI476889 | V | Vietnam | Rust |  |
| 176 | PI476905A | V | China | Rust |  |
| 177 | PI506550A | V | Japan | Oil |  |
| 178 | PI506668 | V | Japan | Oil |  |
| 179 | PI506694 | V | Japan | Oil |  |
| 180 | PI507052 | V | Japan | Oil |  |
| 181 | PI507410 | V | Japan | Oil |  |
| 182 | PI507420 | V | Japan | Oil |  |
| 183 | PI507690 | V | Georgia | Oil |  |
| 184 | PI509096 | V | South Korea | Oil |  |
| 185 | PI518664 | V | United States | Rust |  |
| 186 | PI533654 | V | United States | Yield |  |
| 187 | PI534646 | V | United States | Yield |  |
| 188 | PI548452 | V | North Korea | Phase 1 Seq set |  |
| 189 | PI548696 | V | United States | Phase 1 Seq set |  |
| 190 | PI548987 | V | United States | Oil |  |
| 191 | PI567059 | V | Indonesia | Rust |  |
| 192 | PI567180 | V | Vietnam | Rust |  |
| 193 | PI567346 | V | China | Phase 1 Seq set |  |
| 194 | PI578328B | V | Argentina | Yield |  |
| 195 | PI587648 | V | China | Protein |  |
| 196 | PI593654 | V | United States | Yield |  |
| 197 | PI594643 | V | China | Protein |  |
| 198 | PI594660A | V | China | Protein |  |
| 199 | PI594662B | V | China | Protein |  |
| 200 | PI594781 | V | China | Protein |  |
| 201 | PI594880 | V | China | Phase 1 Seq set |  |
| 202 | PI594922 | V | United States | Oil&Phase 1 Seq set |  |
| 203 | PI598358 | V | United States | Yield&Phase 1 Seq set |  |
| 204 | PI603507 | V | China | Oil |  |
| 205 | PI603576B | V | China | Oil |  |
| 206 | PI603677B | V | China | Oil |  |
| 207 | PI603693A | V | China | Protein |  |
| 208 | PI605773 | V | Vietnam | Rust |  |
| 209 | PI605774 | V | Vietnam | Rust |  |
| 210 | PI605824A | V | Vietnam | Rust |  |
| 211 | PI605829 | V | Vietnam | Rust |  |
| 212 | PI605830B | V | Vietnam | Protein |  |
| 213 | PI605838 | V | Vietnam | Rust |  |
| 214 | PI605854B | V | Vietnam | Rust |  |
| 215 | PI605865B | V | Vietnam | Rust |  |
| 216 | PI605877A | V | Vietnam | Protein |  |
| 217 | PI605885B | V | Vietnam | Rust |  |
| 218 | PI605891A | V | Vietnam | Rust |  |
| 219 | PI606364 | V | Vietnam | Protein |  |
| 220 | PI606397B | V | Vietnam | Rust |  |
| 221 | PI606405 | V | Vietnam | Rust |  |
| 222 | PI615445 | V | Vietnam | Rust |  |
| 223 | PI615488 | V | Vietnam | Rust |  |
| 224 | PI615582 | V | United States | Rust |  |
| 225 | PI632418 | V | United States | Phase 1 Seq set |  |
| 226 | PI632637 | V | Vietnam | Rust |  |
| 227 | PI632641B | V | Vietnam | Rust |  |
| 228 | PI632643 | V | Vietnam | Rust |  |
| 229 | PI632654 | V | Vietnam | Rust |  |
| 230 | PI632656A | V | Vietnam | Rust |  |
| 231 | PI632658 | V | Vietnam | Rust |  |
| 232 | PI632663B | V | Vietnam | Rust |  |
| 233 | PI632666 | V | Vietnam | Rust |  |
| 234 | PI632945A | V | Vietnam | Rust |  |
| 235 | PI086490 | VI | Japan | Protein |  |
| 236 | PI090499 | VI | China | Protein |  |
| 237 | PI095969 | VI | South Korea | Oil |  |
| 238 | PI096089 | VI | North Korea | Protein |  |
| 239 | PI123440 | VI | Myanmar | Protein & Phase 1 Seq set |  |
| 240 | PI165673 | VI | China | Protein |  |
| 241 | PI170892 | VI | South Africa | Protein |  |
| 242 | PI171436 | VI | China | Protein |  |
| 243 | PI171437 | VI | China | Protein |  |
| 244 | PI171440 | VI | China | Protein |  |
| 245 | PI174862 | VI | India | Protein |  |
| 246 | PI175187 | VI | India | Protein |  |
| 247 | PI200461 | VI | Japan | Protein |  |
| 248 | PI200502 | VI | Japan | Protein&Rust |  |
| 249 | PI212606 | VI | Afghanistan | Protein |  |
| 250 | PI215693 | VI | India | Protein |  |
| 251 | PI221972 | VI | Japan | Protein |  |
| 252 | PI284815 | VI | Malaysia | Protein |  |
| 253 | PI319529 | VI | China | Protein |  |
| 254 | PI319530 | VI | China | Protein |  |
| 255 | PI346301 | VI | India | Protein |  |
| 256 | PI368037 | VI | Taiwan | Protein |  |
| 257 | PI368039 | VI | Taiwan | Rust |  |
| 258 | PI371609 | VI | Pakistan | Protein |  |
| 259 | PI374219 | VI | South Africa | Yield |  |
| 260 | PI377576 | VI | Taiwan | Protein |  |
| 261 | PI379620 | VI | Taiwan | Protein |  |
| 262 | PI379621 | VI | Taiwan | Protein&Rust |  |
| 263 | PI381683 | VI | Uganda | Protein |  |
| 264 | PI398220 | VI | South Korea | Protein |  |
| 265 | PI398434 | VI | South Korea | Protein |  |
| 266 | PI398479 | VI | South Korea | Protein |  |
| 267 | PI398580 | VI | South Korea | Protein |  |
| 268 | PI398592 | VI | South Korea | Protein |  |
| 269 | PI398606 | VI | South Korea | Protein |  |
| 270 | PI398635 | VI | South Korea | Protein |  |
| 271 | PI398789 | VI | South Korea | Protein |  |
| 272 | PI398824 | VI | South Korea | Protein |  |
| 273 | PI398896 | VI | South Korea | Protein |  |
| 274 | PI398973 | VI | South Korea | Protein |  |
| 275 | PI398983 | VI | South Korea | Protein |  |
| 276 | PI399041 | VI | South Korea | Protein |  |
| 277 | PI399104 | VI | South Korea | Protein |  |
| 278 | PI407738 | VI | China | Oil |  |
| 279 | PI407781C | VI | South Korea | Protein |  |
| 280 | PI416767 | VI | Japan | Oil |  |
| 281 | PI416927 | VI | Japan | Oil |  |
| 282 | PI417252 | VI | Japan | Oil |  |
| 283 | PI417405 | VI | Japan | Oil |  |
| 284 | PI417490 | VI | Japan | Oil |  |
| 285 | PI417503 | VI | Brazil | Protein&Rust |  |
| 286 | PI423921 | VI | Japan | Oil |  |
| 287 | PI423986 | VI | Japan | Oil |  |
| 288 | PI458264 | VI | South Korea | Oil |  |
| 289 | PI476897 | VI | Vietnam | Protein&Rust |  |
| 290 | PI476908 | VI | Vietnam | Rust |  |
| 291 | PI506695 | VI | Japan | Rust |  |
| 292 | PI506706 | VI | Japan | Oil |  |
| 293 | PI506714 | VI | Japan | Oil |  |
| 294 | PI506738 | VI | Japan | Oil |  |
| 295 | PI506938 | VI | Japan | Protein&Rust |  |
| 296 | PI506939 | VI | Japan | Rust |  |
| 297 | PI506976 | VI | Japan | Oil |  |
| 298 | PI507007 | VI | Japan | Rust |  |
| 299 | PI507009 | VI | Japan | Rust |  |
| 300 | PI507014 | VI | Japan | Oil |  |
| 301 | PI507036 | VI | Japan | Oil |  |
| 302 | PI507187 | VI | Japan | Oil |  |
| 303 | PI507210 | VI | Japan | Oil |  |
| 304 | PI507231 | VI | Japan | Oil |  |
| 305 | PI507327 | VI | Japan | Oil |  |
| 306 | PI507459 | VI | Japan | Oil |  |
| 307 | PI511813 | VI | United States | Rust |  |
| 308 | PI518296 | VI | Taiwan | Oil |  |
| 309 | PI518727 | VI | China | Phase 1 Seq set |  |
| 310 | PI548656 | VI | United States | Phase 1 Seq set |  |
| 311 | PI548978 | VI | United States | Phase 1 Seq set |  |
| 312 | PI553050 | VI | United States | Oil |  |
| 313 | PI564261 | VI | United States | Yield | Yes |
| 314 | PI566984 | VI | Indonesia | Rust |  |
| 315 | PI567188 | VI | Vietnam | Rust |  |
| 316 | PI567190 | VI | Vietnam | Rust |  |
| 317 | PI576154 | VI | United States | Yield |  |
| 318 | PI576857 | VI | United States | Yield |  |
| 319 | PI578309 | VI | Nepal | Phase 1 Seq set |  |
| 320 | PI587625B | VI | China | Yield |  |
| 321 | PI587880A | VI | China | Rust |  |
| 322 | PI587886 | VI | China | Rust |  |
| 323 | PI592756 | VI | United States | Yield |  |
| 324 | PI593985 | VI | South Korea | Oil |  |
| 325 | PI593986 | VI | South Korea | Oil |  |
| 326 | PI593990 | VI | South Korea | Oil |  |
| 327 | PI605791A | VI | Vietnam | Rust |  |
| 328 | PI605891B | VI | Vietnam | Rust |  |
| 329 | PI613195 | VI | United States | Yield |  |
| 330 | PI615437 | VI | Vietnam | Rust |  |
| 331 | PI615484 | VI | Vietnam | Rust |  |
| 332 | PI615502 | VI | Vietnam | Rust |  |
| 333 | PI617045 | VI | United States | Rust |  |
| 334 | PI628812 | VI | Brazil | Phase 1 Seq set |  |
| 335 | PI632648 | VI | Vietnam | Rust |  |
| 336 | PI632650 | VI | Vietnam | Rust&Phase 1 Seq set |  |
| 337 | PI632668 | VI | Vietnam | Rust |  |
| 338 | PI632944B | VI | Vietnam | Rust |  |
| 339 | PI635999 | VI | Unknown | Rust |  |
| 340 | PI200492 | VII | Japan | Rust |  |
| 341 | PI424131 | VII | Zimbabwe | Oil |  |
| 342 | PI518295 | VII | Taiwan | Rust |  |
| 343 | PI531068 | VII | United States | Oil |  |
| 344 | PI548490 | VII | Taiwan | Phase 1 Seq set |  |
| 345 | PI561381 | VII | China | Rust |  |
| 346 | PI567016 | VII | Indonesia | Protein |  |
| 347 | PI567039 | VII | Indonesia | Rust |  |
| 348 | PI567046C | VII | Indonesia | Rust |  |
| 349 | PI567068A | VII | Indonesia | Rust |  |
| 350 | PI567076 | VII | Indonesia | Rust |  |
| 351 | PI584506 | VII | United States | Rust |  |
| 352 | PI587905 | VII | China | Rust |  |
| 353 | PI594502 | VII | China | Protein |  |
| 354 | PI594717B | VII | China | Protein |  |
| 355 | PI594740B | VII | China | Protein |  |
| 356 | PI594751A | VII | China | Protein |  |
| 357 | PI594751B | VII | China | Protein |  |
| 358 | PI594751C | VII | China | Protein |  |
| 359 | PI594759D | VII | China | Protein |  |
| 360 | PI594834B | VII | Unknown | Protein |  |
| 361 | PI159925 | VIII | Peru | Phase 1 Seq set |  |
| 362 | PI189402 | VIII | Guatemala | Protein |  |
| 363 | PI197182 | VIII | Malaysia | Rust |  |
| 364 | PI200456 | VIII | Japan | Rust | Yes |
| 365 | PI200488 | VIII | Japan | Rust | Yes |
| 366 | PI200490 | VIII | Japan | Rust |  |
| 367 | PI200523 | VIII | Japan | Oil&Rust |  |
| 368 | PI203398 | VIII | Brazil | Protein&Rust |  |
| 369 | PI203406 | VIII | South Africa | Protein |  |
| 370 | PI204331 | VIII | Suriname | Oil |  |
| 371 | PI205903 | VIII | Thailand | Protein |  |
| 372 | PI205907 | VIII | Thailand | Protein |  |
| 373 | PI239235 | VIII | Thailand | Protein |  |
| 374 | PI281888 | VIII | Indonesia | Oil |  |
| 375 | PI285095 | VIII | Venezuela | Oil | Yes |
| 376 | PI376069 | VIII | Cameroon | Protein |  |
| 377 | PI378693A | VIII | Japan | Protein | Yes |
| 378 | PI416826A | VIII | Japan | Rust |  |
| 379 | PI416873A | VIII | Japan | Rust | Yes |
| 380 | PI416886 | VIII | Japan | Rust | Yes |
| 381 | PI417013 | VIII | Japan | Rust |  |
| 382 | PI417120 | VIII | Japan | Protein&Rust |  |
| 383 | PI417126 | VIII | Japan | Rust |  |
| 384 | PI417134 | VIII | Japan | Rust |  |
| 385 | PI417500 | VIII | Brazil | Phase 1 Seq set |  |
| 386 | PI423959 | VIII | Japan | Rust |  |
| 387 | PI423962 | VIII | Japan | Rust |  |
| 388 | PI441352 | VIII | Indonesia | Protein |  |
| 389 | PI462312 | VIII | India | Rust |  |
| 390 | PI548443 | VIII | China | Protein |  |
| 391 | PI548447 | VIII | China | Phase 1 Seq set |  |
| 392 | PI548479 | VIII | Taiwan | Phase 1 Seq set |  |
| 393 | PI548970 | VIII | United States | Oil |  |
| 394 | PI566961 | VIII | Indonesia | Protein |  |
| 395 | PI566975 | VIII | Indonesia | Rust |  |
| 396 | PI566985B | VIII | Taiwan | Protein |  |
| 397 | PI566987A | VIII | Indonesia | Protein |  |
| 398 | PI566988A | VIII | Indonesia | Rust |  |
| 399 | PI566998A | VIII | Indonesia | Protein |  |
| 400 | PI566998B | VIII | Indonesia | Protein |  |
| 401 | PI567007B | VIII | Indonesia | Protein |  |
| 402 | PI567014A | VIII | Indonesia | Protein |  |
| 403 | PI567017A | VIII | Indonesia | Protein |  |
| 404 | PI567020A | VIII | Indonesia | Rust |  |
| 405 | PI567024 | VIII | Indonesia | Rust |  |
| 406 | PI567025A | VIII | Indonesia | Rust |  |
| 407 | PI567034 | VIII | Indonesia | Rust |  |
| 408 | PI567046A | VIII | Indonesia | Rust |  |
| 409 | PI567054B | VIII | Indonesia | Protein |  |
| 410 | PI567056A | VIII | Indonesia | Rust |  |
| 411 | PI567061 | VIII | Indonesia | Rust |  |
| 412 | PI567066 | VIII | Indonesia | Protein | Yes |
| 413 | PI567069A | VIII | Indonesia | Protein&Rust |  |
| 414 | PI567085B | VIII | Indonesia | Protein |  |
| 415 | PI567108A | VIII | Indonesia | Protein |  |
| 416 | PI567108B | VIII | Indonesia | Protein |  |
| 417 | PI567111 | VIII | Indonesia | Protein |  |
| 418 | PI567117A | VIII | Indonesia | Protein |  |
| 419 | PI567123A | VIII | Indonesia | Rust |  |
| 420 | PI567231 | VIII | China | Phase 1 Seq set | Yes |
| 421 | PI578457A | VIII | Vietnam | Rust |  |
| 422 | PI594307 | VIII | Japan | Phase 1 Seq set |  |
| 423 | PI594433C | VIII | China | Oil |  |
| 424 | PI594470B | VIII | China | Protein |  |
| 425 | PI594470C | VIII | China | Protein |  |
| 426 | PI594511C | VIII | China | Oil |  |
| 427 | PI594721 | VIII | China | Protein |  |
| 428 | PI594760A | VIII | China | Protein |  |
| 429 | PI594839A | VIII | China | Oil |  |
| 430 | PI603537C | VIII | China | Oil | Yes |
| 431 | PI603722 | VIII | China | Protein & Phase 1 Seq set |  |
| 432 | PI603953 | VIII | United States | Oil |  |
| 433 | PI605778B | VIII | Vietnam | Protein |  |
| 434 | PI285090 | IX | Venezuela | Oil |  |
| 435 | PI340900B | IX | Thailand | Protein |  |
| 436 | PI416810 | IX | Japan | Rust |  |
| 437 | PI417080 | IX | Japan | Oil | Yes |
| 438 | PI417085 | IX | Japan | Rust |  |
| 439 | PI417089A | IX | Japan | Rust |  |
| 440 | PI417089B | IX | Japan | Rust | Yes |
| 441 | PI423961A | IX | Japan | Oil&Rust |  |
| 442 | PI428692 | IX | India | Oil |  |
| 443 | PI471900 | IX | Indonesia | Protein |  |
| 444 | PI471904 | IX | Indonesia | Rust |  |
| 445 | PI548973 | IX | United States | Oil | Yes |
| 446 | PI566956 | IX | Indonesia | Rust |  |
| 447 | PI566983 | IX | Indonesia | Protein |  |
| 448 | PI566987B | IX | Indonesia | Protein |  |
| 449 | PI566988B | IX | Indonesia | Protein |  |
| 450 | PI566997 | IX | Indonesia | Protein |  |
| 451 | PI567005A | IX | Indonesia | Protein | Yes |
| 452 | PI567005B | IX | Indonesia | Protein | Yes |
| 453 | PI567005C | IX | Indonesia | Protein |  |
| 454 | PI567006B | IX | Indonesia | Protein |  |
| 455 | PI567007C | IX | Indonesia | Oil |  |
| 456 | PI567012 | IX | Indonesia | Protein |  |
| 457 | PI567025B | IX | Indonesia | Protein |  |
| 458 | PI567040 | IX | Indonesia | Oil |  |
| 459 | PI567049B | IX | Indonesia | Oil |  |
| 460 | PI567052 | IX | Indonesia | Protein |  |
| 461 | PI567053 | IX | Indonesia | Rust |  |
| 462 | PI567058D | IX | Indonesia | Protein&Rust |  |
| 463 | PI567074B | IX | Indonesia | Phase 1 Seq set |  |
| 464 | PI567077B | IX | Indonesia | Protein |  |
| 465 | PI567080 | IX | Indonesia | Protein |  |
| 466 | PI567083C | IX | Indonesia | Oil |  |
| 467 | PI567083D | IX | Indonesia | Protein |  |
| 468 | PI567090 | IX | Indonesia | Rust |  |
| 469 | PI567099A | IX | Indonesia | Rust |  |
| 470 | PI567101 | IX | Indonesia | Oil |  |
| 471 | PI567102B | IX | Indonesia | Rust |  |
| 472 | PI567103 | IX | Indonesia | Protein |  |
| 473 | PI567104B | IX | Indonesia | Rust |  |
| 474 | PI567108C | IX | Indonesia | Protein |  |
| 475 | PI567122D | IX | Indonesia | Protein |  |
| 476 | PI567128A | IX | Indonesia | Protein |  |
| 477 | PI567129 | IX | Indonesia | Protein&Rust |  |
| 478 | PI567137B | IX | Indonesia | Protein |  |
| 479 | PI163308 | X | India | Protein & Oil | Yes |
| 480 | PI238108 | X | Japan | Protein & Oil | Yes |
| 481 | PI281895A | X | Indonesia | Protein & Oil |  |
| 482 | PI307858 | X | India | Protein & Oil |  |
| 483 | PI307866 | X | India | Protein & Oil |  |
| 484 | PI307869 | X | India | Protein & Oil | Yes |
| 485 | PI307880C | X | India | Protein & Oil | Yes |
| 486 | PI307884A | X | India | Protein & Oil | Yes |
| 487 | PI307884B | X | India | Protein & Oil | Yes |
| 488 | PI307888A | X | India | Protein & Oil | Yes |
| 489 | PI307888B | X | India | Protein & Oil | Yes |
| 490 | PI307898 | X | India | Protein & Oil | Yes |
| 491 | PI374170 | X | India | Protein & Oil | Yes |
| 492 | PI374207 | X | India | Phase 1 Seq set | Yes |

Supplemental Table 2. Mean square estimates of an analysis of variance for R1, R3, R8, Reproductive Period, RP, 100-seed weight SDWT, Plant Height (HT) and Harvest weight, assessed in 141 early (E) maturing soybean genotypes, 197 mid (M) maturing soybean genotypes, and 128 late maturing soybean genotypes grown in 2017 A, 2017 B, 2018 A, 2018 B BUG, 2018 B NYA and 2018 B RUB in Rwanda. ***, ** and * Significant at the 0.001, 0.01, and 0.05 probability level. C.V, coefficient of variation, *H^2^*, broad sense heritability on an entry-mean basis. S.D, standard deviation.

| **Test** | **Parameter** | **Genotype (G)** | **Environment (E)** | **G*E** | **Env(Rep)** | **Residuals** | **Range** | **Mean** | **C.V** | ***H^2^*** | **S.D** |
| --- | --- | --- | --- | --- | --- | --- | --- | --- | --- | --- | --- |
| E | R1 | 123.7*** | 4731.3*** | 9.3*** | 11.5*** | 3.6 | 23-67 | 36 | 5.2 | 0.93 | 1.9 |
| E | R3 | 133.4*** | 7726.9*** | 6*** | 13.5*** | 3.8 | 31-69 | 43 | 4.6 | 0.96 | 1.9 |
| E | R8 | 198.5*** | 21841.8*** | 55.7*** | 42.4*** | 6.3 | 73-130 | 90 | 2.8 | 0.73 | 2.5 |
| E | RP | 167*** | 12005*** | 58.7*** | 41.8*** | 8.6 | 35-93 | 54 | 5.4 | 0.67 | 2.9 |
| E | RP_3 | 193.7*** | 11372.4*** | 65.5*** | 84.2*** | 10.5 | 26-83 | 48 | 6.8 | 0.67 | 3.2 |
| E | GDD_R1 | 16473*** | 249625*** | 1124*** | 1728*** | 500 | 312-672 | 433 | 5.2 | 0.93 | 22.4 |
| E | GDD_R3 | 18176*** | 94789*** | 777*** | 1831*** | 558 | 400-867 | 511 | 4.6 | 0.96 | 23.6 |
| E | GDD_R8 | 27350*** | 1321363*** | 6652*** | 5800*** | 904 | 841-1432 | 1084 | 2.8 | 0.76 | 30.1 |
| E | GDD_RP | 22638*** | 793451*** | 7044*** | 5996*** | 1209 | 381-1027 | 651 | 5.3 | 0.71 | 34.8 |
| E | GDD_RP_3 | 25960*** | 1462871*** | 7778 | 10790*** | 1490 | 293-964 | 564 | 6.8 | 0.70 | 38.6 |
| E | HT | 1036.9*** | 4623.1*** | 69.1*** | 496*** | 53.2 | 8-32 | 37 | 19.8 | 0.93 | 7.3 |
| E | HW | 26177*** | 401835*** | 4166*** | 2430 | 1898 | 2-500 | 128 | 34.1 | 0.84 | 43.6 |
| E | SDWT | 172.89*** | 746.38*** | 9.66*** | 14.79** | 5.5 | 6-35 | 17 | 14.2 | 0.95 | 2.4 |
| E | Protein | 29.8 *** | 3203.8*** | 4.3*** | 6.5* | 2.4 | 25 - 45 | 37.5 | 4.0 | 0.86 | 1.6 |
| E | Oil | 12.92 *** | 698.6*** | 1.09*** | 2.08** | 0.6 | 12.6 - 22 | 17 | 4.4 | 0.92 | 0.8 |
| M | R1 | 378.3*** | 7511.3*** | 18*** | 35.7*** | 5 | 28-67 | 41 | 5.4 | 0.96 | 2.2 |
| M | R3 | 385.8*** | 13083.5*** | 15.3*** | 45.1*** | 7 | 32-86 | 48 | 5.5 | 0.96 | 2.7 |
| M | R8 | 312*** | 46738*** | 76*** | 103*** | 19 | 77-136 | 95 | 4.6 | 0.83 | 4.4 |
| M | RP | 140.7*** | 20570.6*** | 70.1*** | 69*** | 13 | 22-96 | 54 | 6.7 | 0.54 | 3.6 |
| M | RP_3 | 152.8*** | 23088.3*** | 83.8*** | 86.8*** | 15.8 | 010-86 | 47 | 8.5 | 0.49 | 4.0 |
| M | GDD_R1 | 49904*** | 238594*** | 2025*** | 5276*** | 686 | 302-841 | 487 | 5.3 | 0.96 | 26.2 |
| M | GDD_R3 | 51909*** | 271254**** | 1731*** | 6640*** | 876 | 431-977 | 572 | 5.2 | 0.97 | 29.6 |
| M | GDD_R8 | 57349*** | 1441331*** | 8085*** | 18053*** | 1113 | 779-1484 | 1126 | 2.96 | 0.85 | 33.4 |
| M | GDD_RP | 18323*** | 1226175*** | 8519*** | 13603*** | 1555 | 220-1028 | 639 | 6.17 | 0.56 | 39.4 |
| M | GDD_RP_3 | 19552*** | 2123426*** | 10068*** | 16582*** | 1824 | 121-979 | 544 | 7.84 | 0.51 | 42.7 |
| M | HT | 3027.1*** | 27654.1*** | 161.2*** | 1254.5*** | 103.7 | 11-120 | 45 | 22.6 | 0.95 | 10.2 |
| M | HW | 30323*** | 844971*** | 5679*** | 27333*** | 2902 | 5-700 | 146 | 36.8 | 0.82 | 53.9 |
| M | SDWT | 262.98*** | 949.06*** | 24.1*** | 35.26*** | 6.2 | 3-38 | 16 | 15.4 | 0.95 | 2.5 |
| M | Protein | 36.1*** | 8730.1*** | 4.9*** | 42*** | 3 | 32 - 53 | 43 | 4 | 0.87 | 1.7 |
| M | Oil | 11.9*** | 55595.8*** | 1.4*** | 12*** | 0.7 | 11-25 | 18 | 4.7 | 0.81 | 0.8 |
| L | R1 | 1804.5*** | 9805.3*** | 70.8*** | 13.4* | 6 | 32-98 | 55 | 4.4 | 0.96 | 2.5 |
| L | R3 | 1721.9*** | 16704.5*** | 50.6*** | 16.6* | 12.3 | 39-108 | 62 | 4.4 | 0.97 | 3.5 |
| L | R8 | 918*** | 54689*** | 142*** | 118** | 41 | 81-165 | 112 | 5.7 | 0.88 | 6.4 |
| L | RP | 402.8*** | 22967*** | 161*** | 19.1 | 14.8 | 20-114 | 57 | 6.7 | 0.63 | 3.9 |
| L | RP_3 | 428*** | 34248*** | 637*** | 38 | 92 | 14-132 | 51 | 17.4 | 0.58 | 9.6 |
| L | GDD_R1 | 244552*** | 252825*** | 8350*** | 1888* | 896 | 380-1038 | 650 | 4.6 | 0.97 | 29.9 |
| L | GDD_R3 | 215689*** | 488227*** | 5466*** | 2578* | 1115 | 477-1133 | 730 | 4.57 | 0.98 | 33.4 |
| L | GDD_R8 | 159174*** | 295307*** | 19782*** | 2020 | 1712 | 964-1893 | 1337 | 3 | 0.88 | 41.4 |
| L | GDD_RP | 50826*** | 425794*** | 22381*** | 2891 | 2207 | 438-1253 | 690 | 6.8 | 0.60 | 47.0 |
| L | GDD_RP_3 | 52944*** | 540538*** | 23538*** | 4570 | 2719 | 169-1290 | 608 | 8.57 | 0.60 | 52.1 |
| L | HT | 5403*** | 126962*** | 629*** | 2593*** | 305 | 9-237 | 81 | 21.8 | 0.90 | 17.5 |
| L | HW | 22883*** | 50640*** | 5879*** | 21465*** | 3635 | 2-490 | 141 | 43 | 0.75 | 60.3 |
| L | SDWT | 161.01*** | 803.19*** | 9.84*** | 13.18* | 5.7 | 3-30 | 13 | 18.8 | 0.95 | 2.4 |
| L | Protein | 25.9*** | 3980*** | 2.6*** | 9*** | 1.9 | 34.5 -53.8 | 45 | 3 | 0.90 | 1.4 |
| L | Oil | 10.85*** | 636.8*** | 0.9*** | 2.3*** | 0.6 | 10.9 -2.5 | 17 | 4.6 | 0.93 | 0.8 |

Supplemental Table 3. Associated markers.

| **Trait** | **SNP ID** | **Chr** | **Position (bp)** | **-log(10)P** | **MAF** | **Allele** | **Allelic effect** | **PVE (%)** | **ENV** | **Common ENV / Known QTLs/Genes** |
| --- | --- | --- | --- | --- | --- | --- | --- | --- | --- | --- |
| Days to Flowering (R1) | ss715580576 | 1 | 55,438,203 | 8.43E-07 | 0.22 | C/T | 1.5 | 3.8 | 18B_RUB |  |
|  | ss715583777 | 2 | 6,812,100 | 1.05E-10 | 0.47 | T/C | -1.2 | 11.1 | 17B | *(Zimmer et al., 2021)* |
|  | ss715581055 | 2 | 12,242,977 | 5.55E-11 | 0.36 | T/G | -2.7 | 9.8 | 18B_RUB | 17B,18A, *(Zimmer et al., 2021)* |
|  | ss715593866 | 6 | 20,940,014 | 1.32E-07 | 0.15 | C/T | 1.5 | 10.9 | 17B | *E1, Glyma.06G207800* |
|  | ss715593873 | 6 | 21,143,967 | 2.51E-12 | 0.10 | C/T | 3.2 | 11.0 | 18B_RUB | *E1, Glyma.06G207800* |
|  | ss715594352 | 6 | 40,054,544 | 1.06E-07 | 0.30 | T/C | 1.0 | 2.3 | 18B_BUG |  |
|  | ss715596416 | 7 | 15,539,761 | 4.11E-09 | 0.11 | G/A | 2.6 | 22.5 | 18B_RUB | 18B_BUG |
|  | ss715598192 | 7 | 43,641,983 | 5.79E-13 | 0.21 | A/G | -1.9 | 11.1 | 18B_BUG | 17B,18A, 18B_NYA, 18B_RUB |
|  | ss715601276 | 8 | 2,759,633 | 7.62E-07 | 0.30 | C/A | 1.1 | 2.3 | 18B_BUG |  |
|  | ss715604692 | 9 | 46,603,154 | 3.61E-07 | 0.17 | A/G | -1.6 | 5.1 | 18B_RUB |  |
|  | ss715605017 | 9 | 49,635,975 | 3.38E-07 | 0.42 | C/T | -1.0 | 10.0 | 18B_BUG |  |
|  | ss715610915 | 11 | 5,854,753 | 6.50E-09 | 0.48 | T/G | 1.4 | 4.0 | 18B_RUB |  |
|  | ss715612719 | 12 | 36,996,279 | 1.05E-07 | 0.33 | G/A | 1.1 | 1.5 | 18B_BUG |  |
|  | ss715616337 | 13 | 42,110,725 | 2.63E-07 | 0.49 | A/G | 0.9 | 1.3 | 18B_BUG |  |
|  | ss715618704 | 14 | 416,318 | 2.29E-07 | 0.18 | A/G | -1.5 | 5.6 | 18B_RUB |  |
|  | ss715620580 | 15 | 13,329,373 | 1.02E-06 | 0.37 | A/G | 0.8 | 2.8 | 17B |  |
|  | ss715624321 | 16 | 30,708,368 | 1.23E-06 | 0.22 | C/T | -1.5 | 2.5 | 18B_RUB |  |
|  | ss715624323 | 16 | 30,714,729 | 2.11E-09 | 0.25 | G/A | -1.3 | 7.5 | 17A | *700Kbp upstream of E9, Glyma.16G150700* |
|  | ss715626298 | 17 | 1,730,391 | 3.15E-08 | 0.22 | A/G | -1.6 | 1.2 | 18B_NYA | *700Kbp upstream of E9, Glyma.16G150700* |
|  | ss715626651 | 17 | 2,950,757 | 1.18E-06 | 0.24 | A/G | 2.3 | 0.2 | 18B_RUB |  |
|  | ss715583777 | 2 | 6,812,100 | 2.23E-08 | 0.47 | T/C | -0.9 | 5.3 | AAE | *(Zimmer et al., 2021)* |
|  | ss715581055 | 2 | 12,242,977 | 1.27E-13 | 0.36 | T/G | -1.8 | 13.7 | AAE | *(Zimmer et al., 2021)* |
|  | ss715595959 | 7 | 130,157 | 2.30E-07 | 0.46 | T/C | 0.8 | 2.0 | AAE |  |
|  | ss715596416 | 7 | 15,539,761 | 4.33E-09 | 0.11 | G/A | 1.7 | 3.8 | AAE |  |
|  | ss715598192 | 7 | 43,641,983 | 2.46E-16 | 0.21 | A/G | -1.8 | 13.6 | AAE |  |
|  | ss715602749 | 8 | 8,273,185 | 6.08E-08 | 0.25 | A/C | 0.9 | 2.1 | AAE |  |
|  | ss715610916 | 11 | 5,858,427 | 1.58E-08 | 0.48 | G/T | 0.9 | 2.7 | AAE |  |
|  | ss715614954 | 13 | 30,305,993 | 4.23E-07 | 0.16 | C/T | -0.9 | 0.2 | AAE |  |
|  | ss715626631 | 17 | 2,884,236 | 1.14E-07 | 0.38 | T/C | -1.0 | 2.7 | AAE |  |
|  | ss715637323 | 20 | 32,894,943 | 2.66E-08 | 0.06 | T/C | 1.7 | 23.0 | AAE |  |
| **Trait** | **SNP ID** | **Chr** | **Position (bp)** | **-log(10)P** | **MAF** | **Allele** | **Allelic effect** | **PVE (%)** | **ENV** | **Common ENV / Known QTLs/Genes** |
| Days to beginning pod (R3) | ss715578964 | 1 | 3,099,830 | 3.09E-07 | 0.14 | C/T | -1.1 | 3.2 | 17B |  |
|  | ss715580165 | 1 | 51,769,619 | 4.43E-08 | 0.07 | A/C | -2.4 | 8.0 | 18B_NYA |  |
|  | ss715581872 | 2 | 3,314,326 | 1.75E-07 | 0.21 | G/T | -0.9 | 2.4 | 17B |  |
|  | ss715583777 | 2 | 6,812,100 | 5.80E-08 | 0.44 | T/C | -0.9 | 2.5 | 17B | *(Zimmer et al., 2021)* |
|  | ss715581055 | 2 | 12,242,977 | 2.45E-18 | 0.32 | T/G | -2.4 | 5.1 | 17B | 18B_NYA *(Zimmer et al., 2021)* |
|  | ss715585522 | 3 | 34,875,311 | 5.72E-08 | 0.16 | T/C | -1.0 | 6.6 | 18A |  |
|  | ss715586009 | 3 | 38,986,336 | 1.12E-07 | 0.45 | G/T | -0.8 | 3.8 | 17B |  |
|  | ss715586469 | 3 | 44,080,219 | 6.66E-08 | 0.43 | T/C | -1.2 | 7.7 | 18B_BUG |  |
|  | ss715592826 | 6 | 11,853,957 | 7.55E-08 | 0.25 | G/T | -0.9 | 2.9 | 17B |  |
|  | ss715593866 | 6 | 20,940,014 | 3.83E-12 | 0.16 | C/T | 1.7 | 9.4 | 17B | *E1, Glyma.06G207800* |
|  | ss715594155 | 6 | 33,000,707 | 4.40E-07 | 0.26 | A/C | 1.2 | 8.6 | 18B_BUG |  |
|  | ss715594399 | 6 | 41,856,644 | 1.14E-07 | 0.36 | A/G | 0.9 | 6.3 | 18A |  |
|  | ss715595025 | 6 | 49,303,210 | 1.53E-07 | 0.20 | T/C | -1.1 | 2.1 | 17B |  |
|  | ss715596392 | 7 | 15,392,901 | 6.42E-07 | 0.11 | A/G | 1.3 | 6.2 | 17B |  |
|  | ss715596441 | 7 | 15,658,813 | 8.16E-11 | 0.11 | C/T | 1.8 | 8.0 | 17B |  |
|  | ss715597663 | 7 | 37,804,136 | 6.25E-09 | 0.24 | T/C | -1.0 | 19.0 | 17B |  |
|  | ss715598192 | 7 | 43,641,983 | 6.74E-12 | 0.21 | A/G | -2.3 | 16.1 | 18B_NYA | 18A, 18B_RUB |
|  | ss715610844 | 11 | 5,262,636 | 1.14E-07 | 0.47 | A/G | 0.8 | 3.6 | 17B |  |
|  | ss715620326 | 15 | 11,441,207 | 1.18E-09 | 0.40 | T/C | 1.0 | 2.5 | 17B |  |
|  | ss715626293 | 17 | 1,715,795 | 1.14E-09 | 0.22 | C/T | -1.9 | 0.2 | 18B_RUB | 18A |
|  | ss715628658 | 18 | 12,127,004 | 2.90E-10 | 0.44 | C/T | -1.0 | 5.3 | 17B |  |
|  | ss715634054 | 19 | 33,766,555 | 8.74E-09 | 0.08 | T/C | -2.1 | 26.6 | 18B_BUG |  |
|  | ss715637225 | 20 | 30,744,968 | 1.30E-07 | 0.32 | A/G | -0.9 | 0.0 | 18A |  |
|  | ss715581055 | 2 | 12,242,977 | 2.10E-07 | 0.36 | T/G | -1.4 | 26.9 | AAE | *(Zimmer et al., 2021)* |
|  | ss715598192 | 7 | 43,641,983 | 7.59E-12 | 0.21 | A/G | -1.7 | 17.4 | AAE |  |
|  | ss715626293 | 17 | 1,715,795 | 1.41E-06 | 0.22 | C/T | -0.9 | 0.9 | AAE | *(Zimmer et al., 2021)* |
| **Trait** | **SNP ID** | **Chr** | **Position (bp)** | **-log(10)P** | **MAF** | **Allele** | **Allelic effect** | **PVE (%)** | **ENV** | **Common ENV / Known QTLs/Genes** |
| Days to Maturity (R8) | ss715578790 | 1 | 2,506,568 | 4.71E-12 | 0.15 | T/C | -3.1 | 14.2 | 18B_BUG |  |
|  | ss715579430 | 1 | 4,289,439 | 7.58E-07 | 0.17 | A/G | -1.3 | 11.3 | 17A |  |
|  | ss715583627 | 2 | 5,654,643 | 1.19E-08 | 0.28 | C/T | -1.5 | 8.5 | 18B_BUG |  |
|  | ss715583777 | 2 | 6,812,100 | 6.77E-07 | 0.47 | T/C | -1.2 | 1.0 | 18A | *(Zimmer et al., 2021)* |
|  | ss715581055 | 2 | 12,242,977 | 9.71E-08 | 0.35 | T/G | -4.3 | 18.9 | 18B_RUB | *(Zimmer et al., 2021)* |
|  | ss715587062 | 4 | 12,208,154 | 8.91E-07 | 0.42 | G/A | 1.2 | 3.8 | 17B |  |
|  | ss715587091 | 4 | 13,111,309 | 2.94E-08 | 0.19 | C/T | 1.7 | 1.7 | 17B |  |
|  | ss715588448 | 4 | 47,242,528 | 4.19E-07 | 0.13 | T/C | 1.5 | 3.2 | 17B | *(Zimmer et al., 2021)* |
|  | ss715591551 | 5 | 38,038,175 | 8.48E-09 | 0.22 | G/A | -1.7 | 11.4 | 18B_BUG |  |
|  | ss715598498 | 7 | 6,559,823 | 9.65E-09 | 0.45 | T/C | -1.4 | 5.1 | 17B | 18B_BUG |
|  | ss715597660 | 7 | 37,781,401 | 3.21E-08 | 0.22 | C/T | -1.4 | 3.1 | 18A |  |
|  | ss715598192 | 7 | 43,641,983 | 3.64E-11 | 0.21 | A/G | -2.1 | 1.0 | 18A |  |
|  | ss715604243 | 9 | 4,008,493 | 1.14E-06 | 0.19 | G/A | 1.2 | 7.4 | 17A |  |
|  | ss715604542 | 9 | 45,099,143 | 3.04E-08 | 0.16 | G/T | -3.5 | 11.5 | 18B_NYA | Pod maturity 28-5 |
|  | ss715605595 | 10 | 13,532,314 | 2.19E-10 | 0.16 | T/G | -2.0 | 8.4 | 18B_BUG |  |
|  | ss715609217 | 11 | 24,968,419 | 5.77E-07 | 0.12 | C/T | -1.9 | 11.0 | 18B_BUG |  |
|  | ss715613204 | 12 | 5,724,257 | 3.53E-07 | 0.08 | A/G | -1.9 | 6.2 | 17B | ~200 Kbp upstream of *Tof 12, PRR7 (Lu et al., 2020)* |
|  | ss715615357 | 13 | 32,917,293 | 1.01E-06 | 0.48 | A/C | -3.2 | 5.7 | 18B_NYA |  |
|  | ss715620567 | 15 | 13,286,787 | 8.49E-11 | 0.19 | G/A | -1.8 | 21.6 | 17B |  |
|  | ss715624323 | 16 | 30,714,729 | 5.41E-07 | 0.25 | G/A | -1.3 | 0.2 | 18A | *~600 Kbp upstream of E9, Glyma.16G150700* |
|  | ss715626121 | 17 | 13,646,620 | 3.10E-09 | 0.23 | T/C | -3.5 | 30.8 | 18B_NYA |  |
|  | ss715632555 | 18 | 6,752,771 | 2.98E-07 | 0.08 | G/A | -1.9 | 0.7 | 18A |  |
|  | ss715637323 | 20 | 32,894,943 | 9.86E-09 | 0.06 | T/C | 3.4 | 21.3 | 17B |  |
|  | ss715580114 | 1 | 51,309,036 | 5.73E-10 | 0.50 | C/A | 1.2 | 43.8 | AAE |  |
|  | ss715583777 | 2 | 6,812,100 | 8.38E-10 | 0.47 | T/C | -1.3 | 3.1 | AAE | *(Zimmer et al., 2021)* |
|  | ss715581055 | 2 | 12,242,977 | 5.81E-18 | 0.36 | T/G | -2.7 | 5.9 | AAE | *(Zimmer et al., 2021)* |
|  | ss715583075 | 2 | 44,180,381 | 1.44E-08 | 0.32 | C/T | -1.1 | 2.4 | AAE |  |
|  | ss715586469 | 3 | 44,080,219 | 3.62E-10 | 0.43 | T/C | -1.4 | 2.8 | AAE |  |
|  | ss715598550 | 7 | 6,935,933 | 2.14E-08 | 0.14 | G/A | 1.6 | 4.2 | AAE |  |
|  | ss715609113 | 11 | 24,509,473 | 5.61E-07 | 0.20 | G/A | -1.5 | 3.8 | AAE |  |
|  | ss715612855 | 12 | 38,225,497 | 3.26E-07 | 0.11 | T/C | 1.3 | 4.0 | AAE |  |
|  | ss715635456 | 19 | 45,273,019 | 9.98E-07 | 0.33 | T/C | 1.2 | 2.8 | AAE | *Dt1, Glyma.19G194300* |
|  | ss715637321 | 20 | 32,835,139 | 1.94E-07 | 0.11 | A/C | 1.5 | 5.3 | AAE |  |
| **Trait** | **SNP ID** | **Chr** | **Position (bp)** | **-log(10)P** | **MAF** | **Allele** | **Allelic effect** | **PVE (%)** | **ENV** | **Common ENV / Known QTLs/Genes** |
| Reproductive period, Days between First flower and Maturity | ss715579430 | 1 | 4,289,439 | 1.71E-07 | 0.17 | A/G | -1.1 | 12.0 | 17A |  |
|  | ss715583513 | 2 | 48,179,429 | 1.38E-06 | 0.27 | A/C | -1.1 | 3.5 | 18B_BUG |  |
|  | ss715589113 | 4 | 6,109,332 | 2.12E-09 | 0.11 | T/G | 1.7 | 18.3 | 17A |  |
|  | ss715589438 | 4 | 8,553,916 | 2.28E-13 | 0.09 | G/A | 29.6 | 0.8 | 18A | *700 Kbp downstream of E8, Glyma.04g101500* |
|  | ss715589443 | 4 | 8,561,130 | 2.28E-13 | 0.09 | C/T | 29.6 | 1.4 | 18A | *700 Kbp downstream of E8, Glyma.04g101500* |
|  | ss715589462 | 4 | 8,618,416 | 2.28E-13 | 0.09 | G/A | 29.6 | 8.1 | 18A | *700 Kbp downstream of E8, Glyma.04g101500* |
|  | ss715587487 | 4 | 33,719,401 | 7.32E-13 | 0.34 | T/C | 1.5 | 5.1 | 18B_BUG |  |
|  | ss715588236 | 4 | 45,842,325 | 1.25E-06 | 0.09 | T/C | -1.9 | 22.6 | 17A |  |
|  | ss715594754 | 6 | 47,741,090 | 1.31E-06 | 0.23 | G/A | -1.0 | 37.6 | 18A |  |
|  | ss715603753 | 9 | 38,848,463 | 2.73E-07 | 0.33 | A/G | 2.7 | 7.1 | 18B_NYA |  |
|  | ss715607500 | 10 | 45,546,527 | 7.83E-07 | 0.40 | A/G | -2.5 | 5.3 | 18B_NYA | *E2, Glyma.10G221500 (GIGANTEA,G1)* |
|  | ss715610927 | 11 | 5,925,409 | 1.10E-06 | 0.23 | A/G | -1.1 | 4.1 | 18B_BUG | *Tof 12, PRR7 (Lu et al., 2020)* |
|  | ss715613180 | 12 | 5,502,184 | 5.14E-09 | 0.11 | T/G | -4.4 | 22.6 | 18B_RUB |  |
|  | ss715613025 | 12 | 39,848,844 | 3.05E-07 | 0.19 | A/C | -1.4 | 4.4 | 18B_BUG |  |
|  | ss715615357 | 13 | 32,917,293 | 3.88E-09 | 0.48 | A/C | -3.4 | 9.1 | 18B_NYA |  |
|  | ss715621991 | 15 | 4,516,820 | 2.01E-08 | 0.40 | A/G | -1.1 | 2.7 | 18B_BUG |  |
|  | ss715620428 | 15 | 12,338,897 | 1.90E-08 | 0.32 | T/C | 1.2 | 3.4 | 18B_BUG |  |
|  | ss715626581 | 17 | 2,697,863 | 9.69E-10 | 0.23 | G/A | 1.6 | 47.2 | 18B_BUG |  |
|  | ss715628036 | 17 | 5,462,570 | 1.52E-10 | 0.20 | G/A | 1.8 | 5.2 | 18B_BUG |  |
|  | ss715638930 | 20 | 47,708,029 | 9.50E-07 | 0.35 | G/A | -1.0 | 6.0 | 18A |  |
|  | ss715589451 | 4 | 8,588,285 | 2.18E-07 | 0.10 | C/T | -1.3 | 25.2 | AAE | *700 Kbp downstream of E8, Glyma.04g101500* |
|  | ss715591701 | 5 | 41,376,185 | 2.96E-14 | 0.18 | A/G | -2.0 | 16.5 | AAE | *(Zimmer et al., 2021)* |
|  | ss715628330 | 17 | 8,892,748 | 2.09E-07 | 0.37 | T/C | 0.9 | 5.9 | AAE |  |
|  | ss715627738 | 17 | 40,410,264 | 1.98E-11 | 0.43 | T/G | -1.2 | 3.4 | AAE | *(Zimmer et al., 2021)* |
|  | ss715631050 | 18 | 45,278,829 | 3.39E-07 | 0.05 | C/T | -1.9 | 9.1 | AAE |  |
| **Trait** | **SNP ID** | **Chr** | **Position (bp)** | **-log(10)P** | **MAF** | **Allele** | **Allelic effect** | **PVE (%)** | **ENV** | **Common ENV / Known QTLs/Genes** |
| Reproductive period, Days between Beginning Pod and Maturity | ss715589438 | 4 | 8,553,916 | 1.15E-15 | 0.09 | G/A | 31.6 | 17.5 | 18A | *700 Kbp downstream of E8, Glyma.04g101500* |
|  | ss715589443 | 4 | 8,561,130 | 1.15E-15 | 0.09 | C/T | 31.6 | 5.0 | 18A | *700 Kbp downstream of E8, Glyma.04g101500* |
|  | ss715589462 | 4 | 8,618,416 | 1.15E-15 | 0.09 | G/A | 31.6 | 5.4 | 18A | *700 Kbp downstream of E8, Glyma.04g101500* |
|  | ss715604633 | 9 | 4,316,401 | 5.94E-07 | 0.39 | A/G | 0.9 | 3.7 | 18A |  |
|  | ss715603753 | 9 | 38,848,463 | 6.79E-07 | 0.33 | A/G | 2.6 | 8.4 | 18B_NYA |  |
|  | ss715615357 | 13 | 32,917,293 | 4.95E-10 | 0.48 | A/C | -3.7 | 9.7 | 18B_NYA |  |
|  | ss715587823 | 4 | 39,484,148 | 2.68E-08 | 0.28 | T/C | 1.1 | 3.6 | AAE | *1Mbp downstream of E1La, Glyma.04G156400* |
|  | ss715591701 | 5 | 41,376,185 | 1.32E-06 | 0.18 | A/G | -1.2 | 20.0 | AAE | *(Zimmer et al., 2021)* |
|  | ss715622945 | 15 | 6,796,528 | 1.36E-06 | 0.43 | G/A | 0.8 | 26.9 | AAE |  |
| **Trait** | **SNP ID** | **Chr** | **Position (bp)** | **-log(10)P** | **MAF** | **Allele** | **Allelic effect** | **PVE (%)** | **ENV** | **Common ENV / Known QTLs/Genes** |
| Plant Height (cm) | ss715579290 | 1 | 40,427,922 | 8.80E-07 | 0.45 | A/G | -2.7 | 8.3 | 18B_BUG |  |
|  | ss715583777 | 2 | 6,812,100 | 1.38E-06 | 0.47 | T/C | -2.5 | 8.7 | 17B | *(Zimmer et al., 2021)* |
|  | ss715581055 | 2 | 12,242,977 | 4.20E-07 | 0.36 | T/G | -6.0 | 17.4 | 18B_RUB | *(Zimmer et al., 2021)* |
|  | ss715581079 | 2 | 12,410,880 | 3.79E-07 | 0.36 | C/T | 4.7 | 8.5 | 18B_NYA |  |
|  | ss715582839 | 2 | 42,029,552 | 1.62E-07 | 0.09 | G/A | -4.8 | 11.0 | 18A |  |
|  | ss715583075 | 2 | 44,180,381 | 1.16E-09 | 0.33 | C/T | -3.1 | 3.9 | 18B_BUG | 17B, *(Zimmer et al., 2021)* |
|  | ss715585261 | 3 | 32,699,430 | 8.23E-07 | 0.08 | A/G | -4.4 | 15.2 | 18B_BUG |  |
|  | ss715590413 | 5 | 4,597,560 | 8.10E-08 | 0.08 | G/T | -8.9 | 37.1 | 18B_NYA |  |
|  | ss715590979 | 5 | 34,140,084 | 8.40E-07 | 0.19 | T/C | -4.0 | 16.5 | 18B_RUB |  |
|  | ss715609618 | 11 | 2,070,903 | 1.16E-06 | 0.06 | G/A | -4.7 | 10.5 | 18B_BUG |  |
|  | ss715609217 | 11 | 24,968,419 | 3.52E-08 | 0.12 | C/T | -4.5 | 13.8 | 18B_BUG |  |
|  | ss715615575 | 13 | 34,733,930 | 9.52E-08 | 0.29 | A/C | 2.8 | 0.0 | 17A |  |
|  | ss715620101 | 14 | 9,237,374 | 2.47E-08 | 0.27 | A/G | 3.0 | 5.8 | 18B_BUG |  |
|  | ss715617684 | 14 | 13,312,001 | 6.40E-09 | 0.45 | A/G | 2.8 | 5.3 | 17B |  |
|  | ss715620326 | 15 | 11,441,207 | 5.72E-07 | 0.37 | T/C | 2.5 | 6.0 | 18B_BUG |  |
|  | ss715620618 | 15 | 13,610,877 | 3.39E-07 | 0.36 | T/C | -2.6 | 9.4 | 17B |  |
|  | ss715624580 | 16 | 3,281,243 | 1.40E-07 | 0.28 | G/A | 2.4 | 10.6 | 17B |  |
|  | ss715628792 | 18 | 12,928,558 | 1.32E-06 | 0.33 | G/T | 5.8 | 7.0 | 18B_NYA |  |
|  | ss715629792 | 18 | 20,419,500 | 1.03E-06 | 0.50 | T/G | 2.5 | 5.9 | 18A |  |
|  | ss715635425 | 19 | 45,204,441 | 1.77E-07 | 0.33 | A/C | 3.4 | 10.5 | 17B | *Dt1, Glyma.19G194300* |
|  | ss715583777 | 2 | 6,812,100 | 2.76E-11 | 0.47 | T/C | -2.9 | 12.9 | AAE | *(Zimmer et al., 2021)* |
|  | ss715581055 | 2 | 12,242,977 | 2.97E-11 | 0.36 | T/G | -4.1 | 5.8 | AAE | *(Zimmer et al., 2021)* |
|  | ss715582839 | 2 | 42,029,552 | 3.03E-11 | 0.09 | G/A | -4.8 | 9.2 | AAE |  |
|  | ss715583075 | 2 | 44,180,381 | 1.98E-09 | 0.32 | C/T | -2.6 | 3.9 | AAE | *(Zimmer et al., 2021)* |
|  | ss715596571 | 7 | 16,641,225 | 8.18E-07 | 0.36 | A/G | -2.1 | 3.7 | AAE |  |
|  | ss715598192 | 7 | 43,641,983 | 1.16E-10 | 0.21 | A/G | -3.6 | 8.2 | AAE |  |
|  | ss715619184 | 14 | 46,010,999 | 3.86E-08 | 0.36 | C/T | -2.7 | 8.1 | AAE |  |
|  | ss715622702 | 15 | 50,955,781 | 1.20E-07 | 0.07 | G/A | 3.8 | 7.1 | AAE |  |
|  | ss715626121 | 17 | 13,646,620 | 1.15E-09 | 0.23 | T/C | -2.8 | 5.3 | AAE |  |
|  | ss715629808 | 18 | 20,486,726 | 1.41E-06 | 0.33 | A/G | 1.9 | 1.9 | AAE |  |
|  | ss715635458 | 19 | 45,292,930 | 1.96E-11 | 0.34 | C/T | 3.6 | 8.0 | AAE | *Dt1, Glyma.19G194300* |
| **Trait** | **SNP ID** | **Chr** | **Position (bp)** | **-log(10)P** | **MAF** | **Allele** | **Allelic effect** | **PVE (%)** | **ENV** | **Common ENV / Known QTLs/Genes** |
| 100-seed weight, g | ss715580637 | 1 | 55,939,194 | 3.34E-08 | 0.17 | T/C | -1.1 | 6.8 | 18B_BUG |  |
|  | ss715584786 | 3 | 2,231,287 | 8.18E-08 | 0.13 | C/T | -1.3 | 10.1 | 18B_BUG |  |
|  | ss715589103 | 4 | 635,354 | 6.56E-07 | 0.42 | G/A | -0.6 | 10.7 | 17B |  |
|  | ss715587073 | 4 | 12,473,858 | 2.77E-07 | 0.49 | C/T | -0.7 | 2.0 | 18B_BUG | *(Yuan et al., 2024)* |
|  | ss715587677 | 4 | 22,602,482 | 2.14E-09 | 0.24 | T/C | 0.8 | 11.0 | 17B |  |
|  | ss715588255 | 4 | 45,977,762 | 1.37E-07 | 0.49 | T/C | 0.7 | 4.1 | 18B_BUG |  |
|  | ss715592223 | 5 | 5,875,471 | 1.50E-15 | 0.08 | T/C | 2.4 | 24.6 | 18B_BUG |  |
|  | ss715598475 | 7 | 6,360,555 | 4.43E-07 | 0.15 | A/G | -0.9 | 11.4 | 17A |  |
|  | ss715598178 | 7 | 43,453,490 | 2.36E-07 | 0.43 | A/G | 0.6 | 7.7 | 17B | 18B_RUB |
|  | ss715602066 | 8 | 43,371,191 | 1.29E-06 | 0.20 | G/A | -0.7 | 4.8 | 18A |  |
|  | ss715603315 | 9 | 2,180,637 | 2.29E-08 | 0.06 | T/C | 1.6 | 16.6 | 18B_RUB |  |
|  | ss715606303 | 10 | 35,024,662 | 1.40E-06 | 0.27 | G/A | 0.6 | 0.4 | 17B |  |
|  | ss715610718 | 11 | 4,339,089 | 6.68E-07 | 0.31 | A/G | -0.7 | 18.1 | 18A |  |
|  | ss715611002 | 11 | 6,523,082 | 2.72E-07 | 0.25 | G/A | -0.8 | 17.4 | 18B_NYA |  |
|  | ss715609855 | 11 | 17,540,742 | 4.95E-09 | 0.41 | T/C | 1.1 | 11.6 | 18B_BUG |  |
|  | ss715609824 | 11 | 18,538,596 | 5.61E-08 | 0.39 | A/G | 0.7 | 10.1 | 17B |  |
|  | ss715613833 | 13 | 20,471,963 | 1.08E-06 | 0.15 | T/C | -1.0 | 20.4 | 18B_RUB |  |
|  | ss715619776 | 14 | 6,561,966 | 1.88E-07 | 0.45 | G/A | -0.7 | 0.0 | 18A |  |
|  | ss715618217 | 14 | 33,571,207 | 1.93E-12 | 0.26 | A/C | 1.3 | 8.3 | 18B_BUG |  |
|  | ss715620758 | 15 | 14,405,482 | 2.20E-08 | 0.24 | T/C | -0.7 | 10.0 | 17B |  |
|  | ss715624152 | 16 | 29,583,982 | 1.40E-07 | 0.25 | C/A | 0.8 | 10.4 | 18B_RUB |  |
|  | ss715626515 | 17 | 2,492,277 | 3.00E-07 | 0.31 | C/T | -0.7 | 10.5 | 18B_NYA |  |
|  | ss715626124 | 17 | 13,676,996 | 2.90E-07 | 0.26 | A/G | 0.8 | 17.1 | 17A |  |
|  | ss715585814 | 3 | 393,566 | 1.23E-06 | 0.36 | G/A | 0.4 | 2.6 | AAE |  |
|  | ss715594173 | 6 | 33,801,116 | 1.26E-08 | 0.07 | G/A | -1.2 | 3.2 | AAE |  |
|  | ss715610853 | 11 | 5,297,684 | 2.83E-08 | 0.16 | A/C | -0.8 | 1.9 | AAE |  |
|  | ss715609824 | 11 | 18,538,596 | 3.80E-08 | 0.39 | A/G | 0.7 | 11.7 | AAE |  |
|  | ss715612136 | 12 | 3,215,512 | 3.16E-07 | 0.30 | G/T | -0.5 | 3.3 | AAE |  |
|  | ss715611894 | 12 | 21,203,953 | 4.23E-07 | 0.09 | A/G | 0.8 | 5.8 | AAE |  |
|  | ss715618217 | 14 | 33,571,207 | 8.73E-10 | 0.26 | A/C | 0.7 | 16.5 | AAE |  |
| **Trait** | **SNP ID** | **Chr** | **Position (bp)** | **-log(10)P** | **MAF** | **Allele** | **Allelic effect** | **PVE (%)** | **ENV** | **Common ENV / Known QTLs/Genes** |
| Single row plot haverst weight, g | ss715592525 | 5 | 715,155 | 3.19E-07 | 0.05 | A/G | -20.2 | 16.9 | 18A |  |
|  | ss715595669 | 6 | 9,401,334 | 6.73E-07 | 0.09 | C/T | 30.2 | 37.3 | 18A |  |
|  | ss715592826 | 6 | 11,853,957 | 6.02E-07 | 0.24 | G/T | -14.4 | 3.9 | 18B_BUG |  |
|  | ss715598297 | 7 | 52,876 | 4.92E-07 | 0.48 | T/C | 10.4 | 5.6 | 18A |  |
|  | ss715595759 | 7 | 10,425,142 | 3.17E-07 | 0.36 | G/A | -16.0 | 3.8 | 17B |  |
|  | ss715597998 | 7 | 40,896,029 | 2.91E-07 | 0.19 | C/T | -17.4 | 21.2 | 17B | 18B_BUG |
|  | ss715605199 | 9 | 5,870,243 | 4.16E-08 | 0.34 | C/T | -14.1 | 4.0 | 18B_BUG |  |
|  | ss715605258 | 9 | 6,196,989 | 1.04E-06 | 0.38 | G/T | -13.0 | 1.0 | 17B |  |
|  | ss715607620 | 10 | 46,730,455 | 1.53E-09 | 0.28 | A/C | 18.7 | 8.3 | 18B_BUG |  |
|  | ss715609748 | 11 | 14,516,640 | 3.29E-08 | 0.19 | C/T | 22.3 | 11.8 | 18B_BUG |  |
|  | ss715609294 | 11 | 25,446,555 | 3.62E-09 | 0.23 | C/T | -14.2 | 6.1 | 18A |  |
|  | ss715614592 | 13 | 28,258,030 | 2.29E-07 | 0.24 | T/C | -13.8 | 3.9 | 18B_BUG |  |
|  | ss715627889 | 17 | 4,040,070 | 3.94E-08 | 0.07 | G/A | -27.4 | 13.7 | 18B_BUG |  |
|  | ss715630151 | 18 | 30,144,818 | 2.02E-08 | 0.41 | T/G | -17.4 | 3.7 | 18B_BUG |  |
|  | ss715634893 | 19 | 39,854,722 | 4.09E-07 | 0.12 | T/C | -18.6 | 5.2 | 18B_BUG |  |
|  | ss715635425 | 19 | 45,204,441 | 1.96E-10 | 0.34 | A/C | 21.5 | 11.0 | 18B_BUG | *Dt1, Glyma.19G194300* |
|  | ss715635774 | 19 | 48,121,741 | 3.05E-07 | 0.36 | T/C | 13.0 | 0.9 | 17B |  |
|  | ss715579327 | 1 | 4,023,442 | 2.96E-08 | 0.29 | G/A | 8.6 | 3.8 | AAE |  |
|  | ss715581493 | 2 | 2,175,799 | 1.81E-10 | 0.38 | C/A | -9.4 | 3.4 | AAE |  |
|  | ss715582592 | 2 | 40,454,101 | 3.08E-10 | 0.12 | A/G | 16.7 | 14.2 | AAE |  |
|  | ss715589613 | 4 | 9,239,829 | 9.05E-08 | 0.32 | G/A | 8.4 | 3.6 | AAE |  |
|  | ss715593889 | 6 | 21,516,983 | 6.36E-13 | 0.31 | A/G | 13.0 | 6.1 | AAE |  |
|  | ss715597998 | 7 | 40,896,029 | 6.62E-08 | 0.19 | C/T | -9.8 | 4.6 | AAE |  |
|  | ss715599497 | 8 | 13,934,839 | 1.29E-08 | 0.29 | G/A | 9.8 | 3.9 | AAE |  |
|  | ss715607043 | 10 | 401,196 | 2.37E-07 | 0.40 | T/C | -7.4 | 1.8 | AAE |  |
|  | ss715609412 | 11 | 27,036,099 | 1.56E-13 | 0.14 | T/G | -15.9 | 12.6 | AAE |  |
|  | ss715623970 | 16 | 28,752,968 | 3.27E-08 | 0.36 | T/G | 9.3 | 5.3 | AAE |  |
|  | ss715634893 | 19 | 39,854,722 | 5.07E-07 | 0.12 | T/C | -9.8 | 7.5 | AAE |  |
|  | ss715635425 | 19 | 45,204,441 | 8.07E-11 | 0.34 | A/C | 12.3 | 6.2 | AAE | *Dt1, Glyma.19G194300* |
| **Trait** | **SNP ID** | **Chr** | **Position (bp)** | **-log(10)P** | **MAF** | **Allele** | **Allelic effect** | **PVE (%)** | **ENV** | **Common ENV / Known QTLs/Genes** |
| Protein (%) | ss715582048 | 2 | 3,790,978 | 1.50E-09 | 0.34 | T/G | -6.5 | 5.5 | 17B |  |
|  | ss715582635 | 2 | 40,700,381 | 9.45E-07 | 0.35 | T/C | -5.4 | 11.8 | 18A |  |
|  | ss715591698 | 5 | 41,404,483 | 1.06E-06 | 0.17 | A/G | 9.6 | 22.7 | 17A |  |
|  | ss715592720 | 6 | 10,851,807 | 1.22E-07 | 0.32 | T/C | 7.3 | 3.4 | 17B |  |
|  | ss715593377 | 6 | 15,576,418 | 1.49E-07 | 0.17 | G/A | -10.1 | 9.2 | 17A |  |
|  | ss715597556 | 7 | 37,173,030 | 3.46E-11 | 0.15 | C/T | -9.9 | 11.9 | 17B |  |
|  | ss715599697 | 8 | 15,326,072 | 8.65E-08 | 0.17 | A/C | -8.9 | 13.5 | 17A |  |
|  | ss715599883 | 8 | 16,954,412 | 9.04E-07 | 0.18 | A/G | -7.7 | 2.9 | 17A |  |
|  | ss715603073 | 9 | 1,477,581 | 2.69E-08 | 0.28 | G/T | 5.7 | 2.8 | 17B |  |
|  | ss715608157 | 10 | 50,645,820 | 4.77E-13 | 0.22 | T/C | 9.8 | 8.7 | 17B |  |
|  | ss715611404 | 12 | 11,604,556 | 9.33E-08 | 0.50 | T/C | -5.6 | 6.3 | 17B |  |
|  | ss715619421 | 14 | 47,621,296 | 6.06E-08 | 0.33 | G/T | -6.2 | 4.8 | 17B |  |
|  | ss715632799 | 18 | 8,553,504 | 4.77E-07 | 0.10 | G/A | -8.6 | 26.0 | 17B |  |
|  | ss715630136 | 18 | 32,273,057 | 1.78E-07 | 0.41 | A/G | 7.0 | 8.7 | 18A |  |
|  | ss715595276 | 6 | 51,029,740 | 6.92E-07 | 0.49 | G/A | -4.0 | 2.6 | AAE |  |
|  | ss715617732 | 14 | 13,567,809 | 2.12E-07 | 0.45 | A/G | 6.3 | 0.0 | AAE |  |
|  | ss715630151 | 18 | 30,144,818 | 1.96E-08 | 0.40 | T/G | 5.8 | 3.8 | AAE |  |
| **Trait** | **SNP ID** | **Chr** | **Position (bp)** | **-log(10)P** | **MAF** | **Allele** | **Allelic effect** | **PVE (%)** | **ENV** | **Common ENV / Known QTLs/Genes** |
| Oil (%) | ss715579065 | 1 | 3,415,315 | 8.89E-09 | 0.25 | G/A | 3.1 | 10.1 | 17A |  |
|  | ss715586315 | 3 | 42,610,920 | 3.74E-07 | 0.22 | C/A | -2.9 | 8.4 | 17A |  |
|  | ss715587263 | 4 | 19,076,840 | 8.85E-08 | 0.21 | A/G | 2.9 | 11.7 | 17B |  |
|  | ss715616625 | 13 | 45,789,704 | 3.42E-08 | 0.06 | A/G | 4.6 | 17.7 | 17B |  |
|  | ss715619247 | 14 | 46,387,888 | 7.12E-08 | 0.20 | A/G | 3.4 | 14.3 | 17A |  |
|  | ss715637283 | 20 | 21,083,902 | 1.20E-09 | 0.17 | A/G | 5.9 | 19.4 | 17B |  |
|  | ss715591642 | 5 | 41,854,786 | 5.54E-07 | 0.32 | C/T | 3.0 | 12.7 | AAE |  |
|  | ss715637294 | 20 | 32,282,623 | 1.96E-07 | 0.20 | T/C | 3.1 | 25.4 | AAE | *POWR1; (Goettel et al., 2022)* |
| **Trait** | **SNP ID** | **Chr** | **Position (bp)** | **-log(10)P** | **MAF** | **Allele** | **Allelic effect** | **PVE (%)** | **ENV** | **Common ENV / Known QTLs/Genes** |
| Growing Degree Days to Flowering, GDD_R1 | ss715583777 | 2 | 6,812,100 | 1.28E-10 | 0.47 | T/C | -15.1 | 11.0 | 17B | *(Zimmer et al., 2021)* |
|  | ss715581055 | 2 | 12,242,977 | 1.32E-09 | 0.36 | T/G | -21.9 | 23.2 | 17B | 18B_BUG, 18B_RUB, *(Zimmer et al., 2021)* |
|  | ss715593866 | 6 | 20,940,014 | 1.27E-08 | 0.15 | C/T | 19.8 | 11.0 | 17B | *E1, Glyma.06G207800* |
|  | ss715594399 | 6 | 41,856,644 | 8.22E-08 | 0.36 | A/G | 11.8 | 2.4 | 18B_BUG | *E1, Glyma.06G207800* |
|  | ss715596892 | 7 | 2,522,750 | 8.40E-08 | 0.12 | C/T | -19.8 | 3.9 | 18B_RUB |  |
|  | ss715598192 | 7 | 43,641,983 | 8.95E-11 | 0.21 | A/G | -20.7 | 8.5 | 18B_BUG | 17B, 18A, 18B_NYA & 18B_RUB |
|  | ss715605017 | 9 | 49,635,975 | 1.22E-07 | 0.42 | C/T | -12.9 | 5.3 | 18B_BUG |  |
|  | ss715610915 | 11 | 5,854,753 | 1.32E-06 | 0.48 | T/G | 11.5 | 6.1 | 18B_RUB |  |
|  | ss715612719 | 12 | 36,996,279 | 7.39E-07 | 0.33 | G/A | 12.7 | 3.6 | 18B_BUG |  |
|  | ss715618704 | 14 | 416,318 | 5.34E-07 | 0.18 | A/G | -14.6 | 5.5 | 18B_RUB |  |
|  | ss715620580 | 15 | 13,329,373 | 5.16E-07 | 0.37 | A/G | 10.6 | 2.8 | 17B |  |
|  | ss715624321 | 16 | 30,708,368 | 1.07E-07 | 0.22 | C/T | -16.6 | 3.1 | 18B_RUB | *700Kbp upstream of E9, Glyma.16G150700* |
|  | ss715624323 | 16 | 30,714,729 | 6.01E-09 | 0.25 | G/A | -15.0 | 10.4 | 17A | *700Kbp upstream of E9, Glyma.16G150700* |
|  | ss715626298 | 17 | 1,730,391 | 2.75E-07 | 0.22 | A/G | -15.9 | 0.9 | 18B_NYA |  |
|  | ss715628232 | 17 | 7,982,457 | 1.02E-06 | 0.07 | G/A | -21.5 | 17.4 | 18B_RUB |  |
|  | ss715634054 | 19 | 33,766,555 | 2.72E-08 | 0.08 | T/C | -22.1 | 19.9 | 18B_BUG |  |
|  | ss715583777 | 2 | 6,812,100 | 2.10E-07 | 0.47 | T/C | -10.1 | 8.6 | AAE | *(Zimmer et al., 2021)* |
|  | ss715581055 | 2 | 12,242,977 | 7.54E-09 | 0.36 | T/G | -19.1 | 15.4 | AAE | *(Zimmer et al., 2021)* |
|  | ss715598192 | 7 | 43,641,983 | 4.09E-13 | 0.21 | A/G | -18.8 | 16.4 | AAE |  |
|  | ss715610916 | 11 | 5,858,427 | 9.82E-07 | 0.48 | G/T | 8.9 | 4.1 | AAE |  |
|  | ss715628565 | 18 | 11,492,415 | 1.08E-06 | 0.23 | C/T | 13.1 | 9.1 | AAE |  |
| **Trait** | **SNP ID** | **Chr** | **Position (bp)** | **-log(10)P** | **MAF** | **Allele** | **Allelic effect** | **PVE (%)** | **ENV** | **Common ENV / Known QTLs/Genes** |
| Growing Degree Days to Beginning Pod, GDD_R3 | ss715583777 | 2 | 6,812,100 | 4.11E-07 | 0.44 | T/C | -11.0 | 9.0 | 17B | *(Zimmer et al., 2021)* |
|  | ss715581055 | 2 | 12,242,977 | 1.19E-08 | 0.32 | T/G | -19.0 | 10.5 | 17B | *(Zimmer et al., 2021)* |
|  | ss715586009 | 3 | 38,986,336 | 4.23E-07 | 0.45 | G/T | -9.5 | 5.3 | 17B |  |
|  | ss715593866 | 6 | 20,940,014 | 9.16E-09 | 0.16 | C/T | 20.2 | 14.2 | 17B | *E1, Glyma.06G207800* |
|  | ss715598192 | 7 | 43,641,983 | 4.23E-11 | 0.21 | A/G | -18.3 | 22.0 | 18A | 18B_NYA, 18B_RUB |
|  | ss715610844 | 11 | 5,262,636 | 1.44E-07 | 0.47 | A/G | 11.3 | 7.2 | 17B |  |
|  | ss715617741 | 14 | 13,587,812 | 1.79E-07 | 0.42 | A/G | 11.6 | 2.6 | 17B |  |
|  | ss715625426 | 16 | 7,396,328 | 5.74E-07 | 0.48 | A/G | -12.0 | 3.0 | 18B_NYA |  |
|  | ss715626293 | 17 | 1,715,795 | 3.16E-09 | 0.22 | C/T | -14.3 | 0 | 18A | 18B_RUB |
|  | ss715628658 | 18 | 12,127,004 | 3.06E-08 | 0.44 | C/T | -11.7 | 12.2 | 17B |  |
|  | ss715629653 | 18 | 19,561,518 | 5.41E-07 | 0.38 | G/A | 4.9 | 10.8 | 18A |  |
|  | ss715634054 | 19 | 33,766,555 | 9.55E-09 | 0.08 | T/C | -25.7 | 33.1 | 18B_BUG |  |
|  | ss715637225 | 20 | 30,744,968 | 1.80E-07 | 0.32 | A/G | -12.5 | 0 | 18A |  |
|  | ss715637323 | 20 | 32,894,943 | 7.25E-10 | 0.06 | T/C | 33.3 | 25.3 | 18B_NYA |  |
|  | ss715581055 | 2 | 12,242,977 | 7.15E-09 | 0.36 | T/G | -18.8 | 27.2 | AAE | *(Zimmer et al., 2021)* |
|  | ss715585505 | 3 | 34,724,265 | 7.17E-08 | 0.18 | T/C | -13.5 | 5.6 | AAE |  |
|  | ss715596892 | 7 | 2,522,750 | 1.05E-09 | 0.12 | C/T | -20.5 | 1.2 | AAE |  |
|  | ss715598192 | 7 | 43,641,983 | 3.53E-12 | 0.21 | A/G | -20.8 | 15.1 | AAE |  |
|  | ss715631536 | 18 | 49,806,593 | 6.02E-08 | 0.30 | T/C | -11.4 | 0.5 | AAE |  |
| **Trait** | **SNP ID** | **Chr** | **Position (bp)** | **-log(10)P** | **MAF** | **Allele** | **Allelic effect** | **PVE (%)** | **ENV** | **Common ENV / Known QTLs/Genes** |
| Growing Degree Days to Maturity, GDD_R8 | ss715579430 | 1 | 4,289,439 | 6.46E-07 | 0.17 | A/G | -17.7 | 11.5 | 17A |  |
|  | ss715583694 | 2 | 6,044,156 | 8.44E-07 | 0.26 | C/T | 14.5 | 1.9 | 18B_BUG |  |
|  | ss715583777 | 2 | 6,812,100 | 9.07E-07 | 0.47 | T/C | -14.2 | 1.0 | 18A |  |
|  | ss715581055 | 2 | 12,242,977 | 7.59E-07 | 0.35 | T/G | -36.7 | 20.3 | 18B_RUB |  |
|  | ss715588448 | 4 | 47,242,528 | 3.40E-07 | 0.13 | T/C | 19.9 | 5.0 | 17B |  |
|  | ss715588458 | 4 | 47,290,025 | 2.28E-07 | 0.27 | T/G | 14.1 | 0.5 | 18A |  |
|  | ss715593996 | 6 | 24,890,301 | 1.12E-06 | 0.08 | A/C | 52.0 | 21 | 18B_RUB |  |
|  | ss715594352 | 6 | 40,054,544 | 1.24E-07 | 0.30 | T/C | 15.8 | 1.2 | 18B_BUG |  |
|  | ss715598498 | 7 | 6,559,823 | 1.43E-06 | 0.45 | T/C | -14.8 | 9.6 | 17B |  |
|  | ss715597660 | 7 | 37,781,401 | 3.32E-08 | 0.22 | C/T | -15.9 | 5.9 | 18A |  |
|  | ss715598192 | 7 | 43,641,983 | 4.45E-11 | 0.21 | A/G | -25.9 | 0.9 | 18A |  |
|  | ss715604243 | 9 | 4,008,493 | 1.02E-06 | 0.19 | G/A | 16.1 | 7.6 | 17A |  |
|  | ss715604985 | 9 | 4,655,784 | 8.59E-09 | 0.38 | C/T | -20.2 | 2 | 18B_BUG | ~500 Kbp upstream of *Tof 9, Glyma.09G056100* |
|  | ss715604542 | 9 | 45,099,143 | 3.55E-08 | 0.16 | G/T | -41.6 | 11 | 18B_NYA |  |
|  | ss715608087 | 10 | 50,156,350 | 1.01E-06 | 0.43 | A/G | -25.4 | 3.3 | 18B_RUB |  |
|  | ss715608759 | 11 | 10,518,944 | 1.24E-07 | 0.12 | G/T | -36.9 | 74.6 | 18B_BUG | *Tof 11, PRR3 (Lu et al., 2020)* |
|  | ss715615357 | 13 | 32,917,293 | 1.06E-06 | 0.48 | A/C | -38.1 | 5.5 | 18B_NYA |  |
|  | ss715624134 | 16 | 29,528,105 | 6.07E-10 | 0.08 | C/T | -45.5 | 4.3 | 18B_BUG |  |
|  | ss715624323 | 16 | 30,714,729 | 9.60E-08 | 0.25 | G/A | -17.9 | 0.3 | 18A | *~700Kbp upstream of E9 (FT2a), Glyma.16G150700* |
|  | ss715626121 | 17 | 13,646,620 | 3.20E-09 | 0.23 | T/C | -41.7 | 33.1 | 18B_NYA |  |
|  | ss715632555 | 18 | 6,752,771 | 6.08E-07 | 0.08 | G/A | -23.2 | 0.6 | 18A |  |
|  | ss715579545 | 1 | 4,576,595 | 2.76E-07 | 0.22 | T/G | 14.2 | 1.5 | AAE |  |
|  | ss715583777 | 2 | 6,812,100 | 7.50E-07 | 0.47 | T/C | -12.7 | 15.0 | AAE |  |
|  | ss715581055 | 2 | 12,242,977 | 3.98E-08 | 0.36 | T/G | -20.3 | 11.1 | AAE |  |
|  | ss715586469 | 3 | 44,080,219 | 2.25E-07 | 0.43 | T/C | -13.9 | 5.7 | AAE |  |
|  | ss715589576 | 4 | 9,078,599 | 2.43E-07 | 0.25 | G/A | -14.9 | 4.0 | AAE | ~300 Kbp downstream of *E8 (CRY1a), Glyma.04g101500* |
|  | ss715588452 | 4 | 47,263,365 | 4.07E-08 | 0.10 | A/G | 20.6 | 3.0 | AAE |  |
|  | ss715598192 | 7 | 43,641,983 | 2.19E-07 | 0.21 | A/G | -18.1 | 7.6 | AAE |  |
|  | ss715627750 | 17 | 40,561,123 | 2.91E-07 | 0.49 | C/A | 16.1 | 17.8 | AAE |  |
|  | ss715637774 | 20 | 36,907,843 | 9.87E-07 | 0.36 | A/G | 12.7 | 2.5 | AAE | ~500 Kbp upstream of *E4 (PHYA2), Glyma.20G090000* |
| **Trait** | **SNP ID** | **Chr** | **Position (bp)** | **-log(10)P** | **MAF** | **Allele** | **Allelic effect** | **PVE (%)** | **ENV** | **Common ENV / Known QTLs/Genes** |
| Growing Degree Days between First flower (R1) and Maturity (R8), Reproductive period (GDD_RP) | ss715579430 | 1 | 4,289,439 | 2.50E-08 | 0.17 | A/G | -15.2 | 10.7 | 17A |  |
|  | ss715583513 | 2 | 48,179,429 | 2.00E-07 | 0.27 | A/C | -15.0 | 4.9 | 18B_BUG |  |
|  | ss715586425 | 3 | 43,484,650 | 1.02E-07 | 0.46 | G/A | 27.7 | 24.8 | 18B_RUB |  |
|  | ss715589113 | 4 | 6,109,332 | 1.28E-10 | 0.11 | T/G | 24.1 | 25.8 | 17A |  |
|  | ss715589438 | 4 | 8,553,916 | 3.73E-11 | 0.09 | G/A | 327.9 | 13.6 | 18A | *700 Kbp downstream of E8, Glyma.04g101500* |
|  | ss715589443 | 4 | 8,561,130 | 3.73E-11 | 0.09 | C/T | 327.9 | 0.0 | 18A | *700 Kbp downstream of E8, Glyma.04g101500* |
|  | ss715589462 | 4 | 8,618,416 | 3.73E-11 | 0.09 | G/A | 327.9 | 1.7 | 18A | *700 Kbp downstream of E8, Glyma.04g101500* |
|  | ss715587487 | 4 | 33,719,401 | 3.77E-11 | 0.34 | T/C | 17.6 | 4.3 | 18B_BUG |  |
|  | ss715603753 | 9 | 38,848,463 | 1.71E-07 | 0.33 | A/G | 32.3 | 9.1 | 18B_NYA |  |
|  | ss715613180 | 12 | 5,502,184 | 1.36E-09 | 0.11 | T/G | -44.3 | 17.6 | 18B_RUB | *Tof 12, PRR7 (Lu et al., 2020)* |
|  | ss715613025 | 12 | 39,848,844 | 1.15E-06 | 0.19 | A/C | -17.7 | 5.0 | 18B_BUG |  |
|  | ss715615357 | 13 | 32,917,293 | 1.07E-09 | 0.48 | A/C | -41.9 | 9.2 | 18B_NYA |  |
|  | ss715616047 | 13 | 38,634,656 | 4.02E-07 | 0.30 | C/A | -27.2 | 5.0 | 18B_RUB |  |
|  | ss715621988 | 15 | 4,513,355 | 1.73E-07 | 0.40 | T/G | -12.8 | 2.4 | 18B_BUG |  |
|  | ss715621104 | 15 | 16,932,966 | 2.04E-07 | 0.06 | G/A | -23.0 | 19.7 | 17A |  |
|  | ss715626581 | 17 | 2,697,863 | 2.45E-08 | 0.23 | G/A | 19.1 | 54.0 | 18B_BUG |  |
|  | ss715628036 | 17 | 5,462,570 | 1.47E-09 | 0.20 | G/A | 21.2 | 4.3 | 18B_BUG |  |
|  | ss715637021 | 20 | 24,889,477 | 5.73E-08 | 0.05 | A/G | -22.9 | 33.4 | 18A |  |
|  | ss715589451 | 4 | 8,588,285 | 4.83E-12 | 0.10 | C/T | -22.4 | 43.2 | AAE |  |
|  | ss715591701 | 5 | 41,376,185 | 3.35E-12 | 0.18 | A/G | -21.6 | 5.3 | AAE | *(Zimmer et al., 2021)* |
|  | ss715616000 | 13 | 37,951,180 | 1.86E-07 | 0.49 | A/G | 9.9 | 1.2 | AAE |  |
|  | ss715594471 | 14 | 4,358,356 | 8.90E-08 | 0.17 | A/G | -12.0 | 3.5 | AAE |  |
|  | ss715628036 | 17 | 5,462,570 | 2.41E-07 | 0.20 | G/A | 13.0 | 5.3 | AAE |  |
|  | ss715631721 | 18 | 51,700,164 | 4.88E-07 | 0.46 | A/G | -10.5 | 3.4 | AAE |  |
| **Trait** | **SNP ID** | **Chr** | **Position (bp)** | **-log(10)P** | **MAF** | **Allele** | **Allelic effect** | **PVE (%)** | **ENV** | **Common ENV / Known QTLs/Genes** |
| Growing Degree Days between Beginning Pod (R3) and Maturity (R8), Reproductive period_3 (RP_3) | ss715578964 | 1 | 3,099,830 | 6.31E-10 | 0.14 | C/T | 23.9 | 82.4 | 17B |  |
|  | ss715589438 | 4 | 8,553,916 | 3.02E-15 | 0.09 | G/A | 397.7 | 16.1 | 18A |  |
|  | ss715589443 | 4 | 8,561,130 | 3.02E-15 | 0.09 | C/T | 397.7 | 6.4 | 18A |  |
|  | ss715589462 | 4 | 8,618,416 | 3.02E-15 | 0.09 | G/A | 397.7 | 5.6 | 18A |  |
|  | ss715604633 | 9 | 4,316,401 | 7.24E-07 | 0.39 | A/G | 11.3 | 3.6 | 18A |  |
|  | ss715603753 | 9 | 38,848,463 | 7.82E-07 | 0.33 | A/G | 31.0 | 8.6 | 18B_NYA |  |
|  | ss715615357 | 13 | 32,917,293 | 3.94E-10 | 0.48 | A/C | -43.1 | 9.8 | 18B_NYA |  |
|  | ss715591701 | 5 | 41,376,185 | 8.87E-11 | 0.18 | A/G | -20.4 | 19.3 | AAE | *(Zimmer et al., 2021)* |
|  | ss715613218 | 12 | 6,018,932 | 8.78E-08 | 0.10 | A/G | -16.7 | 35.1 | AAE |  |
|  | ss715636381 | 19 | 8,345,858 | 8.79E-08 | 0.06 | A/G | 20.8 | 7.7 | AAE |  |
