# Supplementary material for: Genome-wide association study of diverse soybean [Glycine max (L.) Merrill] accessions for agronomic and seed composition traits in Rwanda
Source: Theor Appl Genet. 2026 Sep 26;139(10):281. doi: 10.1007/s00122-026-05383-7 (PMC13616022; doi:10.1007/s00122-026-05383-7)
Supplement: Supplementary file 2 [file 122_2026_5383_MOESM2_ESM.docx]

Supplemental Figure 1. Phylogenetic tree of the 466 germplasm accessions used for this study with 10 Glycine soja accession added as an outgroup.The tree indicated the genetic diversity and countries of origin represented in the panel. Only a subset of the total accessions are indicated by plant introduction number, MG designation, and country of collection.

A.	B.

Supplemental Figure 2. Linkage disequilibrium (LD) decay in euchromatic regions (A) and heterochromatic (B) regions.

Supplemental Figure 3. QQ plots showing results of the genome-wide association mapping for the 466 accessions across maturity groups III to X for several traits (R1, R3, R8, RP, RP_3, Plant Height (PH), Protein, Oil, 100-seed weight and harvest weight (HW)) across all environments.

Supplemental Figure 4. QQ plots showing results of the genome-wide association mapping for the 466 accessions across maturity groups III to X for several traits Growing degree day traits (R1, R3, R8, RP, RP_3) across all environments.
